# Maintaining and updating accurate internal representations of continuous variables with a handful of neurons

**DOI:** 10.1038/s41593-024-01766-5

**Published:** 2024-10-03

**Authors:** Marcella Noorman, Brad K. Hulse, Vivek Jayaraman, Sandro Romani, Ann M. Hermundstad

**Affiliations:** Janelia Research Campus, Howard Hughes Medical Institute, Ashburn, VA USA

**Keywords:** Network models, Dynamical systems, Neural circuits

## Abstract

Many animals rely on persistent internal representations of continuous variables for working memory, navigation, and motor control. Existing theories typically assume that large networks of neurons are required to maintain such representations accurately; networks with few neurons are thought to generate discrete representations. However, analysis of two-photon calcium imaging data from tethered flies walking in darkness suggests that their small head-direction system can maintain a surprisingly continuous and accurate representation. We thus ask whether it is possible for a small network to generate a continuous, rather than discrete, representation of such a variable. We show analytically that even very small networks can be tuned to maintain continuous internal representations, but this comes at the cost of sensitivity to noise and variations in tuning. This work expands the computational repertoire of small networks, and raises the possibility that larger networks could represent more and higher-dimensional variables than previously thought.

## Abstract (web summary)

Many animals rely on internal representations of continuous variables such as head direction to guide behavior. Noorman et al. show how such representations can be accurately maintained in small neural networks, countering decades of theoretical intuition.

## Main

**Fig. 1:**
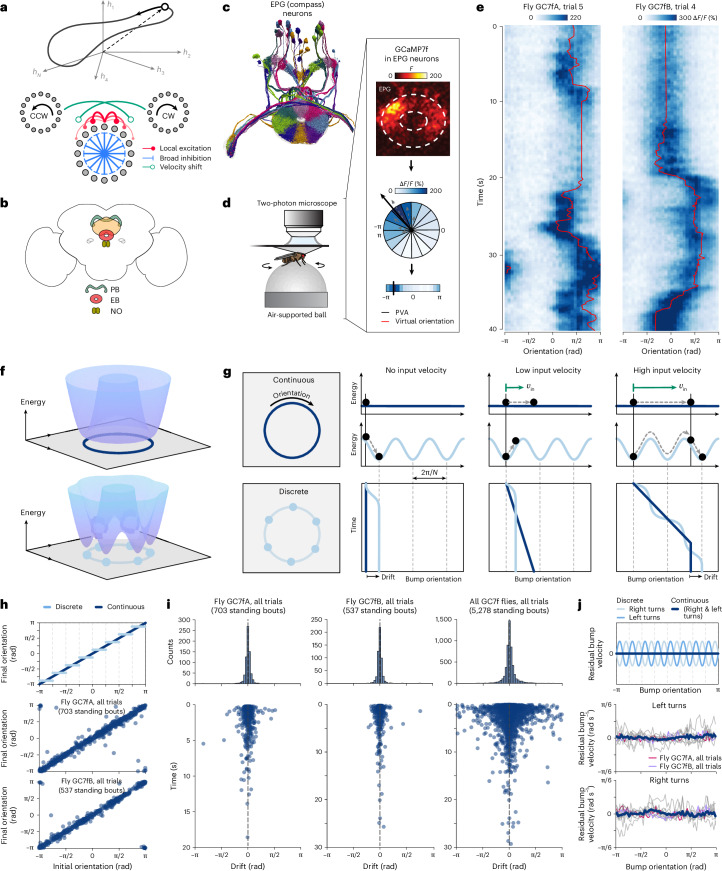
A biological attractor network overcomes hypothesized limitations of discreteness. **a**, Top: ring-like manifold of neural activity. Bottom: a ring attractor network maintains an internal representation of orientation through local excitation (red) and broad inhibition (blue). Two side rings use angular velocity input to shift this representation (green). CW, clockwise; CCW, counterclockwise. **b**, Schematic of the fly CX. ‘Compass’ neurons innervate the EB and maintain an internal representation of orientation. ‘Shift’ neurons innervate the protocerebral bridge (PB) and shift the representation through angular velocity input from the noduli (NO). **c**, Electron microscopy reconstruction of compass neurons. **d**, Two-photon imaging setup for tethered walking flies. Box: 32 regions of interest (ROIs) are used to compute the population vector average (PVA) of the change in fluorescence (Δ*F*/*F*). **e**, Compass neurons maintain a localized bump of activity (heatmap) that tracks the fly’s orientation (red line). **f**, In the absence of input, network dynamics evolve toward the minima of an energy landscape. Infinitely large networks generate flat landscapes (top); small networks generate bumpy landscapes (bottom; illustrated for *N* = 6 neurons). **g**, In continuous networks (dark blue), a flat landscape allows activity to persist at the same orientation in the absence of input (second column) and to integrate velocity input linearly (third and fourth columns). In discrete networks (light blue), local minima cause drift in the absence of input (second column), prevent continuous integration of small inputs (third column), and cause nonlinear integration of large inputs (fourth column). **h**, Bump orientations in the EB before and after stopping periods that exceeded 300 ms, schematized for discrete versus continuous networks (top) and shown for the same flies from **e** (middle and bottom). **i**, Distribution of bump drifts (top histograms) accumulated across stopping periods (bottom scatterplots), shown for the same two flies (left and middle columns) and accumulated across flies (right column). **j**, Residual bump velocities during left versus right turns as a function of bump orientation in the EB, schematized for discrete versus continuous networks (top) and shown for individual flies (middle and bottom; dark blue lines show population averages). Bump velocities were normalized for gain differences before computing residuals (Methods).

The brain is thought to rely on persistent internal representations of continuous variables for a wide range of computations, from working memory^1–4^ to navigation^5–9^ to motor control^10–12^. Such internal representations have been described in terms of manifolds along which population activity evolves (Fig. 1a, top), and they have been studied theoretically within the framework of continuous attractor networks^2,3,5,7,11,13^; see refs. ^14–16^ for recent reviews. This framework for continuous attractor networks has historically relied on large numbers of neurons to ensure that these internal representations are approximately continuous and accurate, and this requirement becomes even more crucial in multiple dimensions and to represent multiple variables. Theories of navigation, for example, rely on large numbers of neurons to explain how continuous attractors could underlie the activity of head direction (HD), place, and grid cells in multiple dimensions^17–19^, and how the hippocampus might build multiple continuous attractors corresponding to different environments that an animal has visited^5,20,21^. Here, we ask whether such continuous representations can be maintained in much smaller networks.

One prominent example of a continuous attractor network is the ring attractor network, which can maintain an internal representation of a periodic variable such as orientation^13,22^, and has been proposed as a model of the HD system^9,23–26^. Ring attractor networks derive their name from the one-dimensional ring manifold on which activity evolves. This manifold emerges in the limit that an infinitely large population of orientation-tuned neurons maintains sustained and localized activity through positive feedback^13,22,24^; this can be achieved through recurrent connectivity by which neurons with similar tuning excite one another, and neurons with dissimilar tuning inhibit one another (Fig. 1a, bottom, and refs. ^13,22,24,27^, but also see ref. ^28^). The resulting population dynamics can generate a localized bump of activity that persists at the same orientation in the absence of input and traverses the ring manifold through the integration of self-motion inputs^23–25^. As a result of their infinite size, ring attractor networks achieve infinite precision in maintaining and accurately updating the bump of activity. Large networks have been used to approximate this infinite precision^2,4,7^; small networks, in contrast, exhibit notable failures that are indicative of finite, rather than infinite, precision^29–31^. Consistent with these studies, we work under the a priori assumption that achieving infinite precision in representing periodic variables requires infinitely large networks (see the Supplementary Note for further discussion).

Although ring attractor networks were proposed theoretically several decades ago, it has been difficult to identify ring-like architectures in brains. Ring attractor networks have been used to explain bell-shaped tuning curves of mammalian HD neurons that display persistent firing in the absence of input and whose activity is updated by self-motion even in darkness^6,32^, but it has not yet been possible to measure patterns of connectivity between these neurons. Mammalian HD neurons have been observed to coherently change their tuning when animals are placed in different settings^6^, and recent work suggests that HD population dynamics traverse a one-dimensional ring-like manifold^33^. In the fly *Drosophila melanogaster*, a recurrent network of neurons in a brain region called the central complex (CX; Fig. 1b) was recently shown to exhibit the functional and structural connectivity^34–36^ (Fig. 1c), as well as the dynamics^8,30,34,37^ (Fig. 1d,e), of a ring-like attractor network. These dynamics are observable as a bump of population activity in so-called EPG or ‘compass’ neurons in a toroidal structure of the CX called the ellipsoid body (EB). This bump of activity tracks the fly’s orientation during turns and persists when the fly stops moving (Fig. 1e). These dynamics are driven both by localizing sensory cues and by the integration of self-motion cues, which enables the bump to track the fly’s movements even in darkness^8,30,37^. The underlying circuit architecture features two subpopulations of ‘shift’ neurons that are jointly tuned to orientation and angular velocity and that receive input from and project back to the compass neurons^30,35–37^, as previously hypothesized^23^ (Fig. 1a, bottom). Thus, both physiological and anatomical considerations suggest that this circuit exhibits the key features of a ring-like attractor network, with one major exception: the fly circuit has far fewer computational units—sets of neurons with the same HD tuning—than are thought necessary to approximate an accurate ring attractor^36^. This low number is likely conserved across many insects, including those that are considered more accomplished navigators, such as bees^38^, suggesting that it does not limit navigational performance. Motivated by these observations, we sought to characterize the capabilities of small networks to represent and integrate an analog, periodic variable. In what follows, we dissect the functional properties of discrete ring-like attractor networks, and show how small circuits might overcome limitations of discreteness to achieve functional performance thought to emerge only in the limit of large systems.

## Results

The computational properties that make ring attractor networks such appealing models of the HD system arise in the limit of large system sizes. Specifically, in the limit that the number of neurons approaches infinity (what we term a ‘continuous’ system), a ring attractor network generates a continuum of configurations that define the ring attractor manifold^13,22,24^ (Fig. 1f, top). These configurations are marginally stable, such that perturbations along the manifold will be maintained, and perturbations off the manifold will be driven back to it. These properties allow us to express the manifold as a flat dimension in the energy landscape of the system^7^; all points along this flat dimension have equal and minimum energy; thus, the system can stably sit at any of these points in the absence of input (Fig. 1g, second column, dark blue). Moreover, small changes in input can drive the system along this flat dimension without obstruction, such that the population activity accurately tracks these changes^23–25^ (Fig. 1g, third and fourth columns, dark blue). This flat energy dimension gives the system infinite precision in encoding and updating an internal representation of a one-dimensional circular variable such as HD.

However, when the system is small (what we term a ‘discrete’ system), these properties are thought to break down, thereby limiting how precisely the internal HD representation can be stored and updated. Instead of exhibiting a flat dimension, the energy landscape is assumed to exhibit a set of discrete basins (Fig. 1f, bottom) that attract the population activity in the absence of input^39^ (Fig. 1g, second column, light blue), prevent the integration of small inputs^14^ (Fig. 1g, third column, light blue), and prevent the accurate integration of large inputs (Fig. 1g, fourth column, light blue). For a small network such as the fly compass network, we would thus expect to observe three distinct signatures of discreteness: (1) drift in the absence of input, in which the HD bump drifts to stereotyped orientations around the EB when the fly stops turning; (2) failure to integrate small angular velocities, in which the HD bump does not move continuously when the fly makes slow turns; and (3) variable responses to larger angular velocities, in which the HD bump moves faster or slower relative to the fly’s movements, depending on its orientation within the EB.

**Extended Data Fig. 1:**
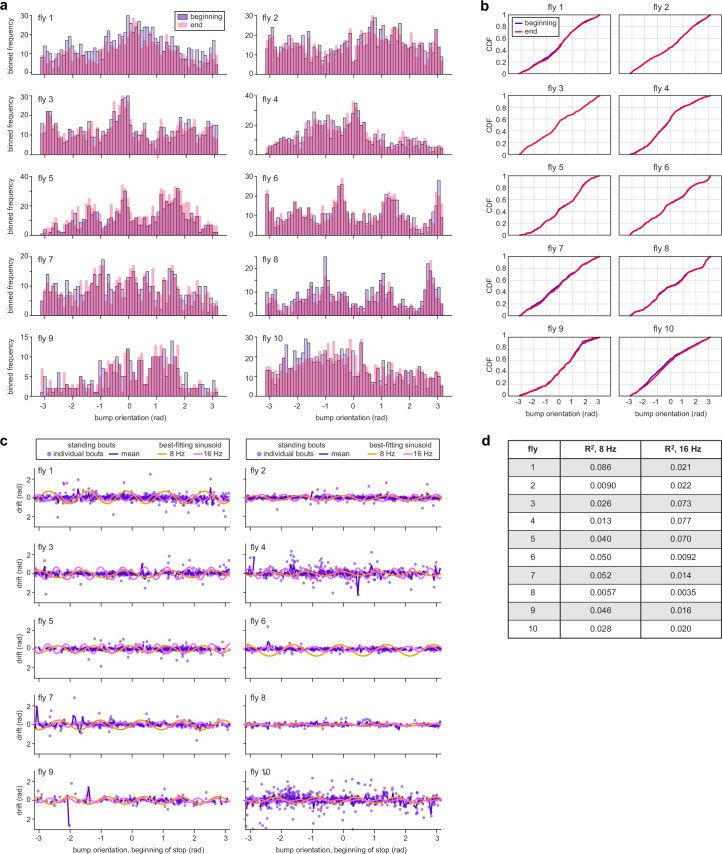
Analysis of bump drift during standing bouts. **a**, Histograms of orientations in the ellipsoid body occupied by the compass bump at the beginning (blue) and end (red) of standing bouts for all ten flies. Note degree of overlap in the distributions, with no sign of an increase in specific orientations from beginning to restart. **b**, Cumulative distributions of orientations in the ellipsoid body occupied by the compass bump at the beginning (blue) and end (red) of standing bouts for all ten flies. Differences between the two distributions are not statistically significant. P-values for Watson’s *U*^2^ test (flies 1-10): 0.5560, 1.0000, 1.0000, 0.9920, 0.9980, 1.0000, 0.9580, 1.0000, 0.9860, 0.1180. *U*^2^ test statistic (flies 1-10): 0.0660, 0.0085, 0.0095, 0.0193, 0.0157, 0.0070, 0.0295, 0.0128, 0.0221, 0.1394. **c**, Drift during standing bouts for all ten flies, measured at different starting orientations of the compass bump. 8- and 16-Hz sinusoids were fit to drifts for each fly. One signature of discreteness in the performance of the compass system would be lower drift when the bump starts at stable orientations during standing bouts and higher drift when the bump starts outside of those orientations. We did not see such fluctuations in the data (see panel **d**). **d**, *R*^2^ values for sinusoidal fits in panel **c**. In panels **a-d**, only those standing bouts that were greater that 0.3 s and less than 2 s were used for analyses. This resulted in the following numbers of standing bouts for flies 1-10: 980, 1005, 835, 826, 723, 714, 573, 527, 312, 949. Flies 2 and 6 correspond to flies GC7fA and GC7fB, respectively, in Fig. 1e,h–j.

**Extended Data Fig. 2:**
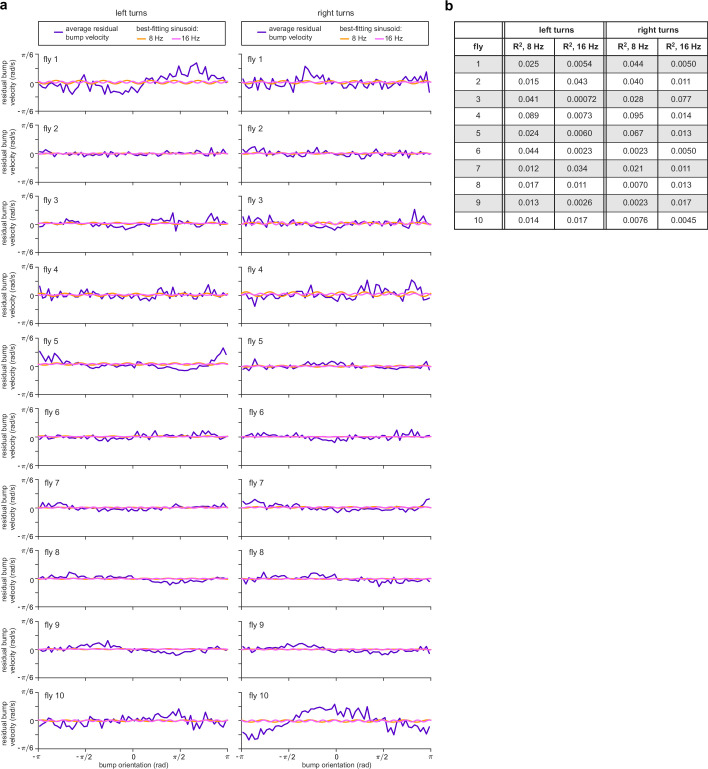
Analysis of residual bump velocity. **a**, Average residual bump velocities measured at different bump orientations, shown separately for left and right turns for all ten flies. 8- and 16-Hz sinusoids were fit to these average residuals. One signature of discreteness would be systematically higher or lower residual velocities at specific bump orientations; we did not see such fluctuations in the data (see panel **b**). **b**, *R*^2^ values for sinusoidal fits in panel **a**.

To assess whether the fly circuit can overcome these expected limitations, we performed two-photon calcium imaging of compass neurons in the EB while head-fixed flies walked on an air-supported ball in darkness (Fig. 1d,e,h–j and Methods). While fly-to-fly variability in the accuracy of integration may be due, in part, to limitations of the fly-on-a-ball system (Methods), several flies showed a remarkable ability to track changes in their angular orientation in darkness. We first measured bump drift in the absence of input^8^ by comparing the bump orientation when the fly stopped moving to when the fly began walking again. The distributions of initial and final bump orientations were similar (Extended Data Fig. 1), and there were no apparent signatures that the bump drifted to a discrete number of stereotypical orientations (Fig. 1h). The distribution of drifts was strongly peaked at zero (Fig. 1i, top row), and included epochs in which the bump persisted at the same orientation for several seconds^8^ (Fig. 1i, bottom row). We then analyzed the average bump velocity at different orientations as a function of the fly’s average turning velocity. Again, across several flies, the bump velocity was consistent across orientations, with no apparent signatures of nonlinear integration nor apparent failures to track small velocities (Fig. 1j and Extended Data Fig. 2). Thus, despite the imperfections of measuring the accuracy of the HD representation in head-fixed flies on a ball, we found that the peak performance of the HD system belied its small size both in its low drift and in its accurate integration.

### Small networks generate a continuum of stable configurations

**Fig. 2:**
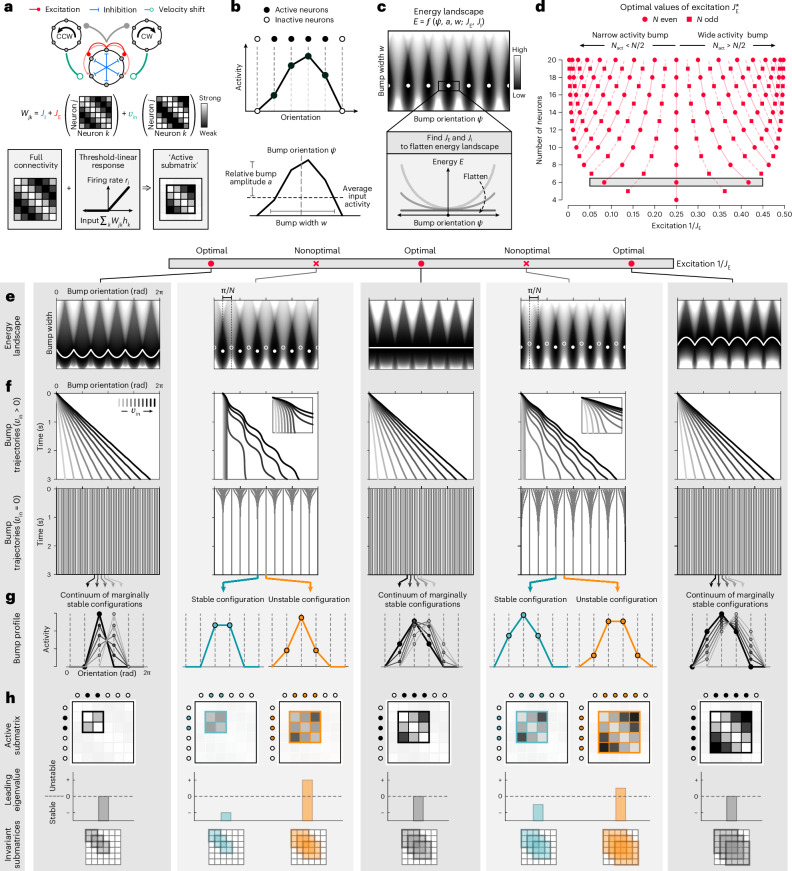
Optimally tuned local excitation can recover a ring attractor manifold. **a**, Schematic of the network model and connectivity *W*_*jk*_. Top: a population of neurons is recurrently connected through local excitation (*J*_E_) and broad inhibition (*J*_I_). Two side rings receive input from and project back to the center ring with shifted, velocity-dependent connections. Bottom: a threshold-linear response function ensures that a subset of *N*_act_ neurons is active at any time; their dynamics are governed by an ‘active submatrix’ of the full connectivity. **b**, Top: *J*_E_ and *J*_I_ can be selected to maintain a persistent bump of population activity. Bottom: characterization of the bump configuration (Methods). **c**, Top: energy of different bump configurations for naive choices of *J*_E_ and *J*_I_. The resulting landscape is bumpy, with local minima (white points) separated by barriers. Bottom: we sought parameters that ‘flatten’ the energy landscape by minimizing local curvature. **d**, For a network of size *N*, there are *N* − 3 optimal values of *J*_E_ that flatten the energy. Shaded bar: optimal values of excitation for a network size of *N* = 6 (see **e**–**h**). **e**–**h**, We evaluate the performance (rows) of networks of size *N* = 6 with different values of *J*_E_ (columns; ${J}_{\textrm{E}}^{* }=[12,4,2.4]$ (optimal); *J*_E_ = [6, 3] (nonoptimal)). **e**, Same as **c**, for different values of *J*_E_. Optimal energy landscapes are flat (white line); nonoptimal landscapes have local minima (filled markers) separated by barriers (open markers). **f**, Bump trajectories in response to a constant input (top row) and in the absence of input (bottom row). Insets show zoomed-in portions of trajectories, which highlight the failure to integrate small inputs. **g**, Same as **b**, shown for bump configurations at the endpoints in **f**. **h**, Top row: same as heatmaps in **a**, shown for active submatrices corresponding to the bump configurations in **g**. Filled markers denote active neurons. Middle row: the leading eigenvalue of each submatrix governs the dynamics of active neurons. Bottom row: in optimal networks, the bump is always maintained by the same number of active neurons (gray); in nonoptimal networks, the bump is maintained by different numbers of active neurons depending on whether the bump configuration is stable (turquoise) or unstable (orange).

The previous results suggest that small networks can, in practice, integrate angular velocity without suffering the performance failures expected of discrete systems. To explore how this might be achieved in principle, we studied the performance of small attractor networks (Fig. 2a and Methods).

We considered networks of *N* orientation-tuned neurons whose preferred orientations *θ*_*j*_ uniformly tile orientation space, with an angular separation of Δ*θ* = 2π/*N* radians (rad). These neurons can be arranged topologically in a ring according to their preferred orientations, with neurons locally exciting and broadly inhibiting their neighbors. We capture this with a symmetric cosine weight matrix ${W}_{jk}^{{\rm{\,sym}}}={J}_{\mathrm{I}}+{J}_{\mathrm{E}}\cos ({\theta }_{j}-{\theta }_{k})$, where *J*_E_ and *J*_I_ respectively control the strength of the tuned and untuned components of recurrent connectivity between neurons with preferred orientations *θ*_*j*_ and *θ*_*k*_. We will refer to these components as local excitation and broad inhibition, respectively (but note that the tuned component takes on both positive and negative values, and thus is not strictly excitatory; within the parameter regimes that we consider, the untuned component is strictly inhibitory). The network receives angular velocity input *v*_in_ through asymmetric, velocity-modulated weights ${W}_{jk}^{{\rm{\,asym}}}=\sin ({\theta }_{j}-{\theta }_{k})$ (see also ref. ^24^); this input could be implemented through two linear side rings whose time constants are much smaller than that of neurons in the center ring (Supplementary Note). Each neuron transforms its inputs through a nonlinear transfer function *ϕ*(⋅). The total input activity *h*_*j*_ of each neuron is then governed by

1 $$\tau {\dot{h}}_{j}=-{h}_{j}+\frac{1}{N}\sum _{k}\left({W}_{jk}^{\;{{\rm{sym}}}}+{v}_{{\rm{in}}}{W}_{jk}^{\;{\rm{asym}}}\right)\phi ({h}_{k})+{c}_{\rm{ff}},\quad j=1,\ldots ,N,$$

where *c*_ff_ is a constant feedforward input to all neurons in the network. In what follows, we take *ϕ*(⋅) to be threshold linear; this ensures that only a subset of all neurons will be active at any time. As a result, the dynamics of active neurons will be governed by an ‘active submatrix’ of the full connectivity (Fig. 2a, bottom). We derive our theoretical results for networks of arbitrary size *N* < ∞; unless otherwise noted, we illustrate these results using a network of size *N* = 6 because this is the smallest network that exhibits the range of dynamics observed across parameter tunings.

**Extended Data Fig. 3:**
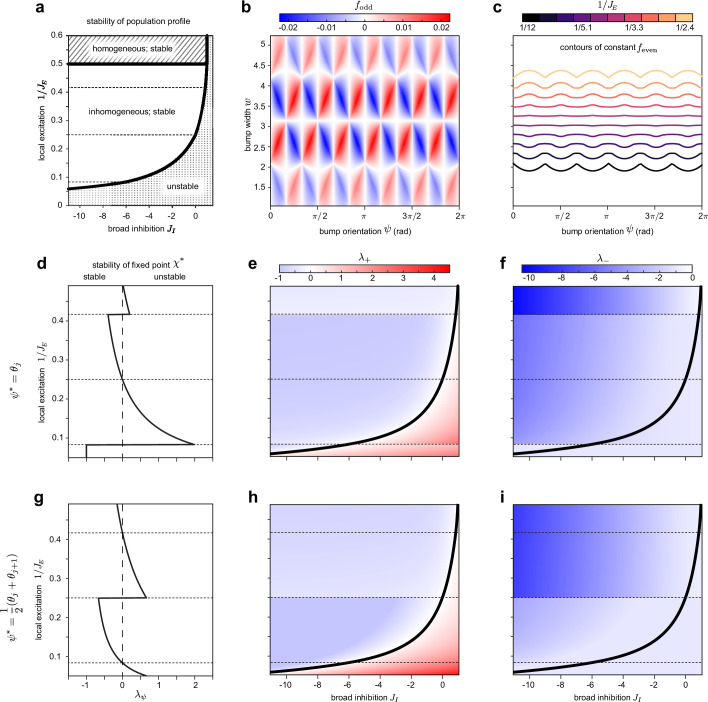
Stability of population profile and fixed-point analysis. **a**, The stability of the shape of the population profile depends on *J*_*E*_ and *J*_*I*_ (shown for *N* = 6). ‘Unstable’ regime: the population activity diverges over time. ‘Homogeneous’ regime: the network generates a stable activity profile that is uniform across the entire network. ‘Inhomogeneous’ regime: the network generates a stable bump of activity that persists at a discrete set of orientations in the absence of input. Dashed lines indicate optimal values of *J*_*E*_ for which the network generates a set of marginally stable solutions that can persist at any orientation in the absence of input. **b-c**, Fixed point conditions from the equations for bump orientation (panel **b**; *f*_odd_ = 0) and relative amplitude (panel **c**; *f*_even_ = 1/*J*_*E*_). See Supplementary Note for details. **b**, Heatmap of *f*_odd_ (*w*, *ψ*) for densely sampled bump widths $w\in \left.\right[2\uppi /N,2(N-1)\pi /N\left.\right)$ and orientations *ψ* ∈ [0, 2π). Red and blue regions correspond to *f*_odd_ > 0 and *f*_odd_ < 0 (which drive the bump orientation to the right and left, respectively). White regions indicate *f*_odd_ = 0, which correspond to potential fixed points ${{\mathcal{X}}}^{* }=({a}^{* },{w}^{* },{\psi }^{* })$ at which the bump can stably persist. Note that at *ψ* = (*θ*_*c*_ + *θ*_*d*_)/2, *d* = *c*, *c* + 1, *f*_odd_ (*w*, *ψ*) = 0 regardless of the value of *w*. **c**, Contours of constant *f*_even_(*w*, *ψ*), shown for 10 evenly spaced values of 1/*J*_*E*_ between and including 1/12 and 1/2.4. These contours indicate a necessary (but not sufficient) relationship between w and *ψ* for stationary bump solutions. **d-i**, Eigenvalues of linearized system about fixed points with orientation ψ^*^ = *θ*_*j*_ (panels **d-f**) or ψ^*^ = (*θ*_*j*_ + *θ*_*j*+1_)/2 (panels **g-i**), *j* = 1…*N*. See Supplementary Note for details. **d**, **g**, Eigenvalue *λ*_*ψ*_ depends on *J*_*E*_. This eigenvalue corresponds to changes in orientation near the fixed points and is the sole determinant of stability of the fixed points. Note that when the set of fixed points corresponding to ψ^*^ = *θ*_*j*_, *j* = 1…*N*, is stable, the other set of fixed points corresponding to ψ^*^ = (*θ*_*j*_ + *θ*_*j*+1_)/2, *j* = 1…*N*, is unstable, and vice-versa. The remaining two eigenvalues *λ*_+_ (panels **e, h**) and *λ*_−_ (panels **f, i**) depend on both *J*_*E*_ and *J*_*I*_ but are always negative in the parameter regime that generates bump-like profiles (region above black line; compare to ‘inhomogeneous; stable’ in panel **a**). Panels **b,c,e,f,h,i** were generated using redblueu.m (https://www.mathworks.com/matlabcentral/fileexchange/74791-redblue-colormap-generator-with-zero-as-white-or-black) and magma.m (https://www.mathworks.com/matlabcentral/fileexchange/51986-perceptually-uniform-colormaps).

For sufficiently strong local excitation and broad inhibition, this network generates a stable bump of activity (Fig. 2b (top), Extended Data Fig. 3a and Methods). We characterize the bump by the Fourier modes of the population activity (given by equation (1)). For the network connectivity chosen here, which varies sinusoidally with the difference between preferred orientations, the population activity is fully specified by the zeroeth- and first-order Fourier modes. This allows us to characterize the ‘configuration’ of the activity bump in terms of its relative amplitude *a*, angular width *w*, and angular orientation *ψ* (Fig. 2b (bottom) and Supplementary Note). These quantities vary continuously over time, and thus, the same number of active neurons can maintain bump configurations with different relative amplitudes, widths, and orientations.

**Extended Data Fig. 4:**
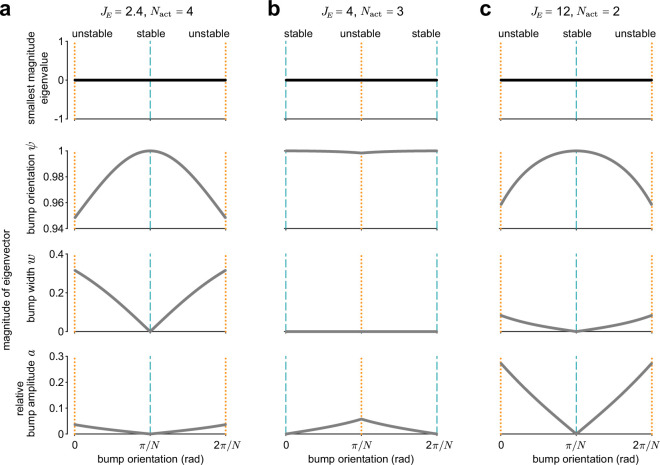
Flat directions in the energy landscape. Smallest magnitude eigenvalues (top row) and corresponding eigenvector components (lower three rows) for the Hessian matrix of the energy, computed for all three optimal values of local excitation in a network of size *N* = 6: **a**, ${J}_{E}^{* }$ = 2.4; **b**, ${J}_{E}^{* }$ = 4; **c**, ${J}_{E}^{* }$ = 12. For each optimal value of local excitation, the Hessian has a single zero eigenvalue, indicating the existence of a zero-curvature direction within the energy landscape. The corresponding eigenvectors are purely aligned along *ψ* (second row) at the orientations of the stable fixed points (teal dashed lines). Away from these orientations, the corresponding eigenvectors involve contributions from *w* and *a* (third and fourth rows, respectively).

We began by characterizing the manifold of stable bump configurations in the absence of angular velocity input (Extended Data Fig. 3b–i and Methods). To this end, we constructed a landscape that describes the energy of different bump configurations for a given set of parameters *J*_E_ and *J*_I_ (refs. ^40,41^ and Methods). For most parameter settings, the energy landscape is bumpy, with discrete minima separated by barriers (Fig. 2c, top), as expected for small networks^39^. The landscape is highly curved about these minima, indicating that the bump would be highly attracted to these particular orientations. To weaken this attraction, we analytically determined the values of *J*_E_ and *J*_I_ that would locally minimize this curvature, and thus locally flatten the energy landscape (Fig. 2c, bottom). Surprisingly, we found that specific values of local excitation drive the curvature to zero, resulting in an energy landscape that is completely flat as a function of orientation (Extended Data Fig. 4). For a network of size *N*, there are *N* − 3 such ‘optimal’ values of local excitation ${J}_{\textrm{E}}^{* }$ (Fig. 2d). Figure 2e illustrates the corresponding optimal energy landscapes for a network of size *N* = 6, and contrasts these with two nonoptimal landscapes generated with intermediate values of local excitation.

**Extended Data Fig. 5:**
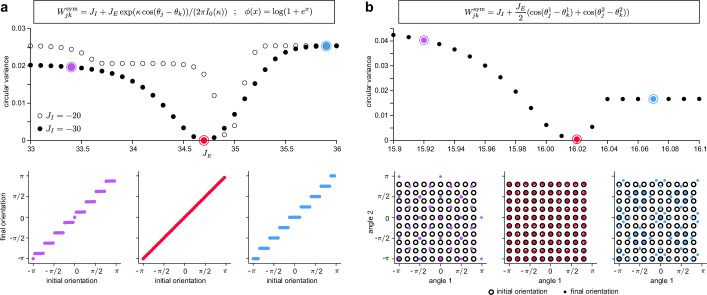
Robustness to changes in the single neuron transfer function and recurrent synaptic weights. Comparison between initial and final bump orientations as a function of *J*_*E*_ for **a**, a network of *N* = 8 neurons with a Von Mises weight profile and a smooth nonlinear transfer function, and **b**, a network of *N* = 16 neurons with a recurrent weight profile storing a 2-dimensional toroidal attractor. In both cases, there is an optimal value of *J*_*E*_ for which the circular variance between the initial and final orientations is close to zero (top, red markers), and the bump does not drift (bottom, center panels). Away from these values of *J*_*E*_, the circular variance increases (top, purple/blue markers), and the bump drifts from its original orientation (bottom left/right panels). See Methods for simulation details.

To verify that these optimally tuned networks could overcome the failure modes highlighted in Fig. 1g, we simulated the response of each network to a constant velocity input (Fig. 2f and Methods). As expected, we found that optimal networks accurately integrated angular velocity input, such that the bump orientation changed linearly over time (Fig. 2f, top row). When this velocity input was removed (Fig. 2f, bottom row), the bump persisted at the same orientation and did not drift (we also observed this in networks with different nonlinearities and connectivity profiles in one and two dimensions; Extended Data Fig. 5 and Methods). In contrast, nonoptimal networks failed to integrate small velocities (Fig. 2f, top row insets), and they nonlinearly integrated larger velocities (Fig. 2f, top row main panels). When this velocity input was removed, the bump drifted toward the set of discrete orientations corresponding to the local minima of their energy landscapes (Fig. 2f, bottom row).

In the absence of velocity input, optimal networks generate a continuum of marginally stable configurations in which the bump can persist (Fig. 2g). These configurations share one striking feature: the bump is always maintained by the same number of active neurons despite variations in relative amplitude, width, and orientation. This feature has important consequences for network dynamics: when a fixed subset of neurons is active, equation (1) for *h*_*j*_ > 0 reduces to a linear dynamical system that depends only on an ‘active submatrix’ of the full connectivity *W* (Fig. 2h, top row; note that we take the full connectivity to be *W* = (*W*^sym^/*N* − *I*)/*τ*). Moreover, because the connectivity is rotationally invariant, this active submatrix—and thus the resulting network dynamics—will be identical for any contiguous subset of *N*_act_ active neurons. To characterize these dynamics, we determined the eigenvalue spectra of these active submatrices (Methods). Each submatrix exhibited a single zero eigenvalue (Fig. 2h, middle row); the real part of all remaining eigenvalues was less than zero. This property gives rise to a so-called line attractor that produces a continuum of marginally stable configurations along a line^11^. Thus, in this network, a ring attractor emerges as a discrete set of *N* line attractors that each governs the dynamics of distinct subsets of active neurons (Fig. 2h, bottom row), and that are ‘stitched together’ at the points where an active subset gains and loses an active neuron.

In contrast, nonoptimal networks can only maintain a discrete set of bump configurations in the absence of input; these configurations correspond to so-called fixed points of the dynamics. One subset of these configurations is stable; the bump will return to these stable fixed points following small perturbations (Fig. 2g, turquoise curves). The other subset is unstable; the bump will move away from these unstable fixed points if perturbed (Fig. 2g, orange curves). In these two configurations—stable and unstable—the bump is maintained by different numbers of active neurons (also called the ‘support’ of the fixed point^42,43^), and the corresponding active submatrices differ in size (Fig. 2h, top row). The smaller of these submatrices has a leading eigenvalue less than zero and governs network dynamics about the stable fixed point, whereas the larger of these submatrices has a leading eigenvalue greater than zero and governs dynamics about the unstable fixed point (Fig. 2h, middle row). In what follows, we use these active submatrices to dissect the dynamics of nonoptimal networks, and we show how the balance between stable and unstable dynamics shapes performance.

### Variations in tuning degrade network performance

**Fig. 3:**
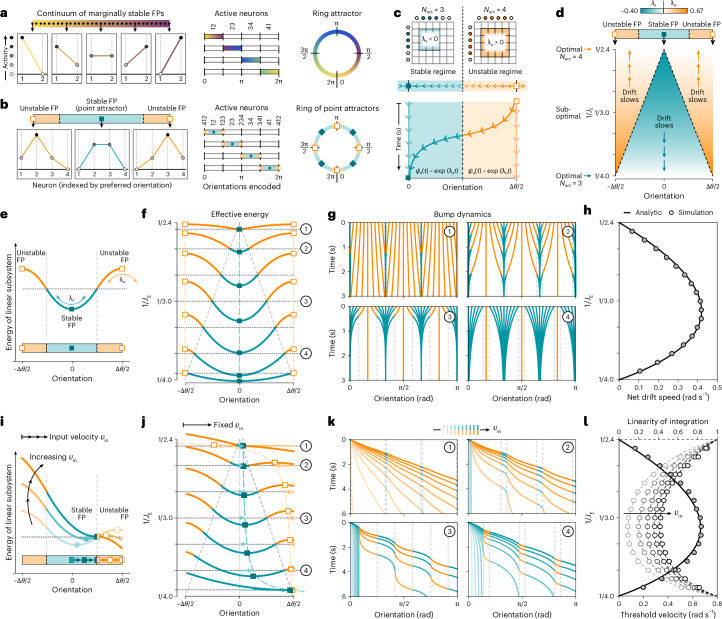
Nonoptimal networks balance periods of stability and instability. **a**, A linear subsystem of active neurons can be tuned to encode a continuum of orientations over a fixed interval (heatmap; left). Multiple line attractors can be stitched together at orientations where the active subset simultaneously gains and loses an active neuron (middle), thereby generating a ring attractor (right). **b**, Without precise tuning, each linear subsystem (shaded region; left) encodes a single unstable or stable fixed point (‘FP’; markers). When stitched together (middle), the set of linear subsystems can stably encode only a finite number of orientations (‘point attractors’; right). **c**, Top: the dynamics of each linear subsystem are governed by the leading eigenvalue *λ* of the active submatrix of the connectivity (Fig. 2h). Bottom: in the unstable regime (orange), the bump accelerates away from an unstable fixed point at rate *λ*_u_ > 0; in the stable regime (turquoise), the bump decelerates toward a stable fixed point at rate *λ*_s_ < 0. **d**, Bump dynamics depend on the fixed-point orientations (square markers), drift rates *λ* (color map), and angular span of each regime (colored areas). Illustrated without velocity input. **e**–**h**, Bump dynamics without velocity input. **e**, Simplified energy landscape. **f**, Same as **e** for different *J*_E_. As *J*_E_ approaches an optimal value, one region of the landscape flattens and fills the entire ring; the other sharpens and shrinks in span. **g**, Bump dynamics for energy landscapes in **f**. **h**, Net drift speed, computed analytically (line) and by simulation (markers). **i**–**l**, Bump dynamics with velocity input. **i**, Small velocities shift the fixed points toward the boundary between stable and unstable regimes, tipping the energy landscape in the direction of the input. At a threshold velocity (equation (5)), the fixed points meet at the boundary, and the bump slides continuously down the landscape. **j**, Same as **i** for different *J*_E_, given a fixed input velocity. *J*_E_ affects how quickly the fixed points move through the energy landscape, and, thus, how readily the landscape tips for a given velocity. **k**, Bump dynamics for energy landscapes in **j**. **l**, Threshold velocity (solid curve) and linearity of integration (dashed curves), computed analytically (lines) and by simulation (markers).

The previous results highlight a unique feature of threshold-linear networks: when a fixed subset of neurons is active, the corresponding dynamical system is linear, and the dynamics of the full network can be viewed as a set of linear subsystems that are stitched together at points where the active subset gains or loses an active neuron. In this way, a ring attractor that encodes a continuum of values on a circle can be constructed by stitching together multiple line attractors that each encode a continuum of values on a line segment. Because a line attractor can be constructed from a network with as few as two neurons, a minimal ring attractor could, in principle, be constructed using only three neurons. However, our choice of connectivity requires a minimum of four neurons to construct a ring attractor, in which each contiguous pair of neurons encodes a distinct line attractor (Fig. 3a). This requires a precise handoff between linear systems that share active neurons, such that the network dynamics move between line attractors by simultaneously activating and inactivating single neurons at the edges of the active subset.

Achieving this precise handoff requires precise tuning, such that the leading eigenvalue *λ* of all active submatrices of *W* is zero. Without a zero eigenvalue, a linear subsystem can, at most, encode a single stable or unstable fixed point. By interleaving linear subsystems that encode stable and unstable fixed points, the network can still cover a circular interval, but the values that can be stably represented are limited to a discrete set (Fig. 3b). In the vicinity of an unstable fixed point (the ‘unstable’ regime), the bump is pushed exponentially quickly away from the fixed point with rate *λ*_u_ > 0 (Fig. 3c, orange). In the vicinity of a stable fixed point (the ‘stable’ regime), the bump is pulled exponentially slowly toward the fixed point with rate *λ*_s_ < 0 (Fig. 3c, turquoise). The bump transitions from the unstable to the stable regime when the active subset loses an active neuron.

This picture highlights how nonlinear computations, such as the integration of angular velocity, can be performed through an orchestrated interaction between multiple linear subsystems that have different fixed-point structures^44^. By decomposing the full dynamical system into linear subsystems, this picture allows us to analytically characterize inaccuracies in nonoptimal networks, and thereby estimate the precision in tuning required to bound these inaccuracies. We measure these inaccuracies using the expected signatures of discreteness highlighted in Fig. 1g (drift in the absence of input, failure to integrate small inputs, and nonlinear integration of large inputs), and we relate these to a simplified description of the energy landscapes shown in Fig. 2e. A complete description of the energy landscape is not attainable in the presence of velocity inputs due to the asymmetry that it introduces in the connectivity matrix (Fig. 2a); to circumvent this, we construct an approximate description that relies on three features of the linear subsystems described above: (1) the orientations of the unstable and stable fixed points, (2) the rates at which the bump is pushed from or pulled toward these fixed points, and (3) the angular span of the regimes governed by each fixed point. As we will show, the local excitation determines the overall curvature of the energy landscape through the rates and angular spans of each regime, which affects the amount of drift. Input velocity shifts the fixed points within this landscape, which influences the accuracy of velocity integration.

#### Drift in the absence of input

**Extended Data Fig. 6:**
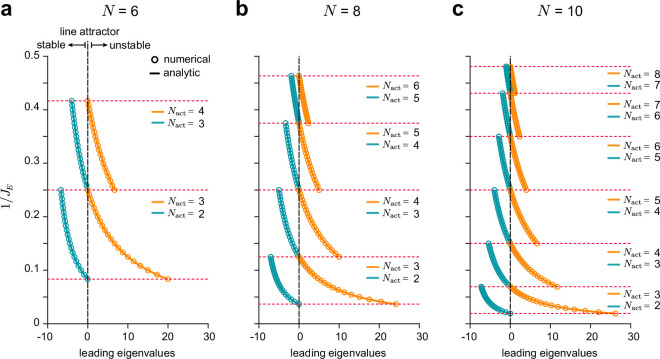
Leading eigenvalues of active submatrices. Comparison of analytically- versus numerically- derived eigenvalues (solid lines versus markers, respectively), computed from the active submatrices of the full connectivity *W* = (*W*^sym^/*N* − *I*)/*τ* in the absence of velocity input. Shown for network sizes **a**, *N* = 6, **b**, *N* = 8, and **c**, *N* = 10. Red dotted lines mark optimal values of local excitation for each network size.

In the absence of velocity input, the stable and unstable fixed points are evenly spaced by Δ*θ*/2 = π/*N* rad regardless of the strength of local excitation. However, the local excitation affects how quickly the bump moves relative to each fixed point, which, in turn, affects the rate of drift in the network. If we vary the local excitation between two optimal values, ${J}_{{\textrm{E}},n}^{* }$ and ${J}_{{\textrm{E}},n+1}^{* }$ (corresponding to scenarios in which the bump is always maintained by *n* or *n* + 1 active neurons, respectively), we find that the drift rates *λ*_s_ and *λ*_u_ depend on how closely tuned the local excitation is to either optimal value (Fig. 3d and Extended Data Fig. 6):

2 $$\begin{array}{rcl}{\lambda }_{\textrm{s}}&=&\left({J}_{\textrm{E}}{\big/}{J}_{{\textrm{E}},n}^{* }-1\right) {\big/}\tau \quad < 0,\\ {\lambda }_{\textrm{u}}&=&\left({J}_{\textrm{E}}{\big /}{J}_{{\textrm{E}},n+1}^{* }-1\right){\big /}\tau > 0.\end{array}$$

Thus, in the stable regime, where the bump is maintained by *n* active neurons, the dynamics depend on how closely tuned the excitation is to the value that would be optimal if *n* neurons maintained the bump. Similarly, in the unstable regime, where the bump is maintained by *n* + 1 active neurons, the dynamics depend on how closely tuned the excitation is to the value that would be optimal if *n* + 1 neurons maintained the bump. Assuming that the bump orientation transitions smoothly between regimes (as seen in simulations; Fig. 2f, top row), the relative widths Δ*θ*_s,u_/Δ*θ* of these regimes depend on the ratio of the drift rates (Fig. 3d):

3 $$\frac{\Delta {\theta }_{\textrm{s}}}{\Delta \theta }=\frac{1}{\left.1+| {\lambda }_{\textrm{s}}| \right/| {\lambda }_{\textrm{u}}| }=1-\frac{\Delta {\theta }_{\textrm{u}}}{\Delta \theta }.$$

Together, these expressions enabled us to construct a simplified landscape that captures the energy of different bump orientations within each linear subsystem (Fig. 3e and Methods). The fixed points determine the locations of extrema within the landscape, the drift rates determine the curvature of the landscape about these extrema, and the angular spans of each regime delineate different regions of the landscape that correspond to stable versus unstable dynamics. This description explains how a ring attractor emerges as the connectivity is tuned toward an optimal value (Fig. 3f): at one extreme (${J}_{\textrm{E}}\to {J}_{{\textrm{E}},n}^{* }$), the stable region of the landscape flattens and expands to fill the entire ring (*λ*_s_ → 0, Δ*θ*_s_ → Δ*θ*), whereas the unstable region sharpens and shrinks in span; at the other extreme (${J}_{\textrm{E}}\to {J}_{{\textrm{E}},n+1}^{* }$), the unstable region of the landscape flattens and expands to fill the entire ring (*λ*_u_ → 0, Δ*θ*_u_ → Δ*θ*), whereas the stable region sharpens and shrinks in span. These differences in the shape of the energy landscape affect the drift dynamics (Fig. 3g), an effect that we quantify by measuring the net drift speed of the bump (Fig. 3h):

4 $$| {\lambda }_{\textrm{d}}| =c\Delta {\theta }_{\textrm{s}}| {\lambda }_{\textrm{s}}| =c\Delta {\theta }_{\textrm{u}}| {\lambda }_{\textrm{u}}| ,$$

where *c* = (e − 1)/2e is a constant. This speed is related to the overall curvature of the landscape, and will be largest at intermediate values of local excitation for which the landscape is bumpiest.

#### Inaccuracies in velocity integration

**Extended Data Fig. 7:**
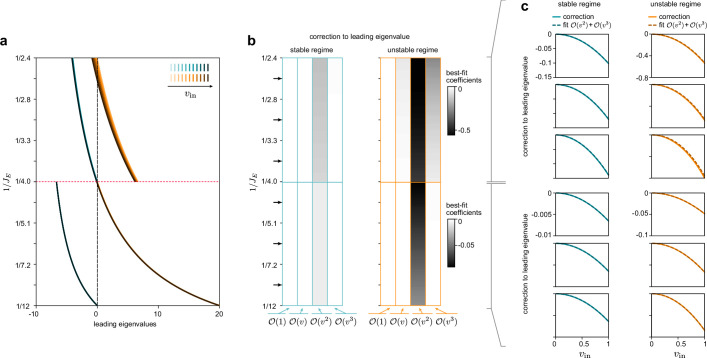
Velocity correction to leading eigenvalues of active submatrices. **a**, Leading eigenvalues *λ* of active submatrices as a function of input velocity *v*_in_ for a network of size *N* = 6. Shown for 11 velocity values evenly spaced between (and including) *v*_in_ = 0 and *v*_in_ = 1 rad s^−^^1^ (darker colors indicate higher velocities). Eigenvalues were obtained by numerically diagonalizing active submatrices of the full connectivity *W* = ((*W*^sym^ + *v*_in_ *W*^asym^)/*N* − *I*)/*τ*. Red dashed line marks an optimal value of local excitation. **b**, Coefficients of the best-fitting 3rd order polynomial of the velocity correction *λ* − *λ*_0_ versus input velocity *v*_in_, where *λ*_0_ is the leading eigenvalue of the full connectivity in the absence of velocity input. **c**, Comparison of the velocity correction *λ* − *λ*_0_ (solid lines) and the best-fitting polynomial (dashed lines), including terms of order *O*(*v*^2^) and *O*(*v*^3^). Shown for 6 different values of local excitation marked by arrows in panel **b**.

**Extended Data Fig. 8:**
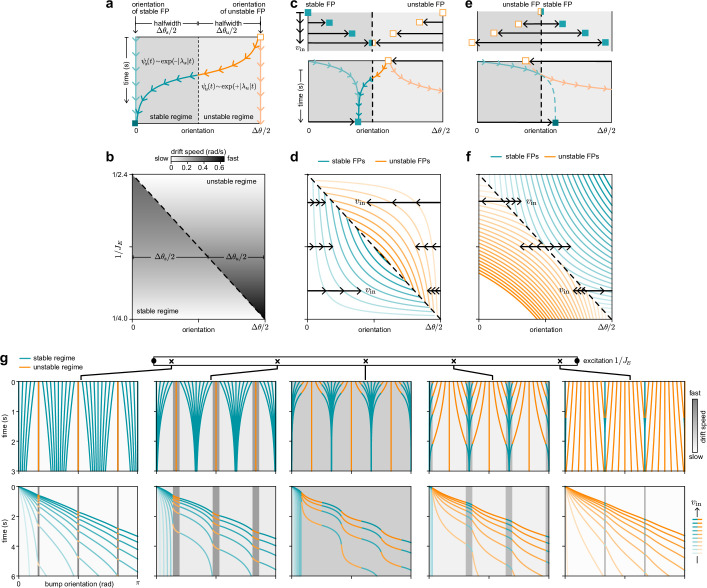
Impact of stable and unstable fixed points on drift and velocity integration. **a-b**, Repeated from Fig. 3c–d, with regimes colored according to drift speed (grayscale). **c**, Input velocity shifts the orientations of the fixed points. Top: as the input velocity increases from 0, the orientations of the stable and unstable fixed points shift toward the boundary between regimes. At a threshold velocity (Eq. (5)), the two fixed points will meet at the boundary; this threshold velocity is the minimum input velocity needed to move the bump continuously. Bottom: For velocities below this threshold, the bump will be driven to the stable fixed point, regardless of its initial orientation. **d**, The orientations of stable and unstable fixed points (turquoise and orange lines, respectively) shift with increasing velocity (darker shades). The rate of these shifts is set by the drift speeds in the stable and unstable regimes (see panel **b**): lower drift speeds lead to faster shifts (marked by the large spacing between turquoise lines at the left of the panel, and between orange lines at the right of the panel). The precise values of these drift speeds ensure that the pair of stable and unstable fixed points will meet at the boundary between regimes at the same threshold velocity, given a fixed value of *J*_*E*_. **e**, Top: as the input velocity increases above the threshold velocity, the stable and unstable fixed points move beyond their respective regimes. Bottom: When in the stable regime, the bump is pulled from ahead toward the stable fixed point. However, before reaching the stable fixed point, the bump transitions into the unstable regime, and is pushed from behind by the unstable fixed point. This push and pull causes the bump to slow down and speed up as it moves through the stable and unstable regimes, respectively; the closer the fixed points are to the boundary, the stronger this effect. **f**, Above the threshold velocity, the stable and unstable fixed points move beyond their respective regimes, and they continue to shift with velocity at the same rate as shown in panel **d**. **g**, Example bump trajectories in the absence (top row) and presence (bottom row) of velocity input.

When a sufficiently small velocity input is injected into the network, the local curvature and angular span of the stable and unstable regions of the landscape will remain approximately unchanged (Extended Data Fig. 7). However, the orientations of the fixed points will shift toward the boundary between regions, thereby tipping the landscape in the direction of the velocity input and driving the bump to a new stable fixed point (Fig. 3i and Extended Data Fig. 8c,d). The flatter the overall landscape (that is, the smaller the value of |*λ*_d_|), the more readily the landscape will tip for a given velocity input (Fig. 3j).

At a particular threshold velocity, *v*_thresh_, the fixed points will meet at the boundary between regions, thereby enabling the bump to slide down the landscape without getting stuck. This threshold velocity specifies the minimum input that can be continuously integrated by the network, and depends on the overall curvature of the landscape through the net drift speed |*λ*_d_|:

5 $${v}_{{\rm{thresh}}}\approxeq \left.| {\lambda }_{\textrm{d}}| \right/2c.$$

The larger the overall curvature of the landscape, the larger the input velocity needed to continuously move the bump (Fig. 3k). In the limit that the local excitation approaches an optimal value, the overall curvature goes to zero, and the network can integrate infinitesimally small inputs (Fig. 3l, solid curve).

Above this threshold velocity, the fixed points will shift outside of their respective regions of the landscape, but their effect will still be felt through the local landscape curvature. As a result, the bump will speed up and slow down as it moves through the unstable and stable regions of the landscape, but it will never get stuck at a fixed point (Fig. 3k and Extended Data Fig. 8e,f). This manifests as nonlinear integration, which we quantify by measuring the ratio between the slowest and fastest bump velocities, ${\nu}_{\min}$ and ${\nu }_{\max }$. This ratio depends only on the relative difference between the threshold and input velocities:

6 $${\rm{linearity}}({v}_{{\rm{in}}})=\frac{{\nu }_{\min }}{{\nu }_{\max }}\approxeq \frac{{v}_{{\rm{in}}}-{v}_{{\rm{thresh}}}}{{v}_{{\rm{in}}}+{v}_{{\rm{thresh}}}}.$$

Bumpier energy landscapes lead to larger threshold velocities, which lead to increasingly nonlinear integration. However, because the overall curvature (and thus the threshold velocity) is fixed for a given value of local excitation, its relative impact on integration decreases as input velocity increases (Fig. 3l, dashed curves). In the limit that the local excitation approaches an optimal value, the threshold velocity goes to zero, and the bump moves continuously at the rate of the input velocity.

### Optimal small networks are less robust

The previous results provide a mechanistic understanding of how small networks can achieve optimal performance through the precise tuning of local excitation. To assess the potential cost of this precision, we used the previous results to characterize how size affects the robustness of optimal networks.

**Fig. 4:**
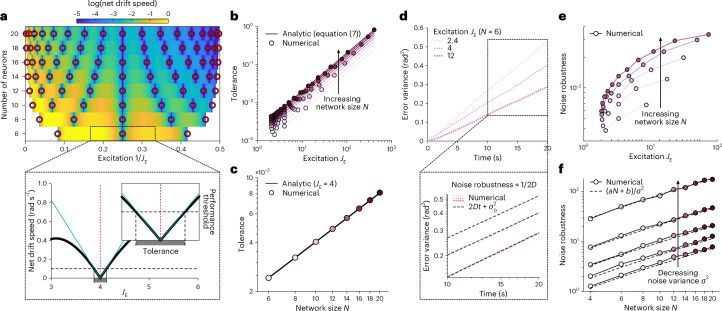
Smaller networks require more fine-tuning and are less robust to noise. **a**, Top: log of net drift speed (color map) as a function of *J*_E_ and *N*. Red circular markers indicate optimal values of ${J}_{\textrm{E}}^{* }$; darker blue colors indicate slower (that is, better) drift rates. Suboptimal networks achieve better performance as *N* increases. Bottom: to estimate tolerance around an optimal value of ${J}_{\textrm{E}}^{* }$, we compute the local change in net drift speed with respect to *J*_E_ (turquoise lines) that will achieve performance below some threshold (horizontal dashed black line, illustrated for a threshold of 0.1 rad s^−1^). **b**, For a given *N* (different colors), larger values of local excitation require less fine-tuning to achieve the same performance. Solid lines mark the analytic tolerance given in equation (7); filled circles indicate the numerically estimated tolerance about each optimal value of ${J}_{\textrm{E}}^{* }$. Results were computed for a threshold value of 0.001 rad s^−1^, and are shown for all evenly sized networks between *N* = 6 and *N* = 20. **c**, Given a fixed value of ${J}_{\textrm{E}}^{* }$, the tolerance increases linearly with *N*. Results are shown for ${J}_{\textrm{E}}^{* }=4$, the only optimal value of local excitation that remains unchanged with even *N*. **d**, Top: error variance between the current and initial bump positions in a small, optimally tuned network with additive Gaussian noise. Numerical results are shown for three different optimal values of ${J}_{\textrm{E}}^{* }$, and with a noise variance *σ*^2^ = (*A*/6)^2^, where *A* = 0.2 is the bump amplitude. Bottom: beyond 10 s, the error variance grows linearly over time, following a diffusion equation with slope 2*D* (where *D* is the diffusion coefficient). We use 1/2*D* as a measure of noise robustness, with lower diffusion signifying higher robustness. **e**, Consistent with **d**, larger optimal values of ${J}_{\textrm{E}}^{* }$ lead to higher noise robustness for a fixed *N*. **f**, Given a fixed value of ${J}_{\textrm{E}}^{* }$ (shown for ${J}_{\textrm{E}}^{* }=4$), noise robustness increases linearly with *N*, and is inversely proportional to noise variance *σ*^2^ (shown for *σ*^2^ = (*A*/6)^2^ × [1, 4, 9, 16, 25]). Dashed lines indicate best linear fits; see Extended Data Fig. 9 for fit coefficients.

**Extended Data Fig. 9:**
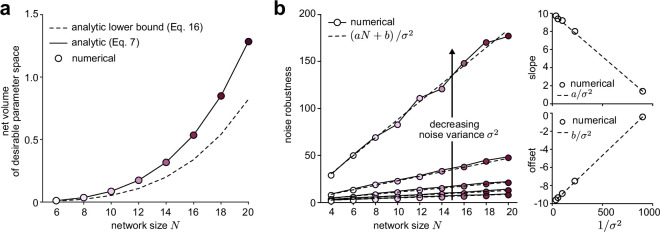
Analysis of robustness as a function of network size. **a**, The net volume of parameter space that achieves a desired performance threshold (estimated by summing the tolerance across all optimal values of local excitation for a given network size *N*) increases faster than *N*^2^. Computed analytically via Eq. (7) by summing over all optimal values of local excitation (solid black line), and estimated numerically by summing over all values shown in Fig. 4b). The analytic lower bound given in Methods Eq. (16) is shown for comparison (gray dashed line). **b**, Left: noise robustness increases linearly with network size. Right: the coefficients of the best linear fit vary inversely with the noise variance *σ*^2^.

We first characterized robustness to variations in parameter tuning. For a given network size, deviations from optimal tuning degrade performance through more rapid drift, larger threshold velocities, and more nonlinear velocity integration. In larger networks, this degradation is less severe (Fig. 4a, top). To quantify this, we asked how precisely the local excitation should be tuned to meet a criterion level of performance (Fig. 4a, bottom). For small values of this criterion, we analytically determined the width of the interval about each optimal value of local excitation ${J}_{\textrm{E}}^{* }$ for which a given measure of network performance meets this criterion; we define the width of this interval to be the tolerance $\,\mathrm{tol}\,(\,{J}_{\textrm{E}}^{* },N\,)$:

7 $$\,\mathrm{tol}\,(\,{J}_{\textrm{E}}^{* },N\,)\ge {c}_{P}{J}_{\textrm{E}}^{* }N,$$

where *c*_*P*_ is a constant that depends on the specific performance measure (net drift rate, threshold velocity, or linearity of integration) and the desired performance criterion. For a given network size, equation (7) shows that larger optimal values of local excitation permit a wider range of parameter values that meet the same criterion level of performance, and are thus more robust to parameter tuning (Fig. 4b). This robustness increases linearly with network size; this can be seen most clearly for ${J}_{\textrm{E}}^{* }=4$, which is an optimal value of local excitation for all evenly sized networks (Fig. 4c). When summed across all optimal values of local excitation, equation (7) allows us to estimate the net volume of parameter space that achieves a desired performance threshold (Methods). Because larger networks permit more values of optimal excitation and exhibit higher tolerances around these values, we find that the net volume of desirable parameter space increases at least quadratically with network size (Extended Data Fig. 9a).

We next characterized robustness to noise. We simulated the dynamics of optimally tuned networks with additive Gaussian noise, and measured how quickly the bump diffused in the absence of velocity input (Fig. 4d, top). At longer timescales, the difference between the initial and final bump positions is diffusive, with a variance that grows linearly over time (Fig. 4d, bottom). The inverse diffusion rate gives a measure of noise robustness; the faster the diffusion, the less robust the network is to noise. For a given network size, larger optimal values of excitation are more robust to noise (Fig. 4e), in qualitative agreement with their increased robustness to variations in parameter tuning (Fig. 4b). For a given value of excitation, noise robustness increases linearly with network size, and inversely with the noise variance (Fig. 4f and Extended Data Fig. 9b).

Together, these results highlight that optimally tuned small networks can recover the performance of infinitely large networks. However, in the networks considered here, this comes at the cost of being less robust to variations in parameter tuning and to noise.

## Discussion

Continuous attractor networks have provided a common theoretical framework for studying a wide range of computations^16^ involved in working memory^2–4^, navigation^5,7,9^, and motor control^11,12^. Across these different task domains, this framework has historically invoked networks of many neurons to ensure smooth and accurate dynamics. However, growing evidence suggests that similar computations might be performed in much smaller brains with far fewer neurons^8,30,34,35,37,45^. Here, we asked to what extent network size limits the performance of attractor networks^3,46^, and whether small networks can overcome these limitations. We focused on a class of attractor networks that maintain a persistent internal representation of a single circular variable, such as orientation, and that update this representation by integrating an internal signal, such as angular velocity. In the limit of infinite numbers of neurons, these ring attractor networks generate a continuous ring manifold along which the population activity smoothly and accurately evolves in the absence of noise. Here, we showed that networks with as few as four neurons could recover this continuous ring attractor manifold, so long as the tuned component of the connectivity (what we term local excitation) is precisely chosen. In the threshold-linear networks studied here, this manifold emerges as a set of line attractor manifolds that govern the dynamics of active subsets of neurons, and that are stitched together to generate a complete ring manifold. The resulting population activity can persist at any orientation in the absence of input, and it can smoothly integrate velocity input.

Together, these results suggest that very small networks can achieve levels of performance that were thought to require large networks. However, this performance comes at the cost of finely tuning local excitation to one of a discrete number of optimal values. Our biological inspiration was the small HD circuit of the fruit fly^8,30,35,37^. Although such networks have been modeled previously^29–31,47^, studies have not demonstrated persistent encoding of arbitrary orientations in the absence of orienting stimuli. Further, although previous studies^31,47^ have shown that network performance changes as connection strengths vary, our study fully characterizes how network size and connection strength influence performance. It is unclear whether the fly HD system relies on the fine-tuning that we require for optimal performance. To date, this system has only been probed under head fixation on an air-supported ball (Methods); thus, its performance during free behavior is unknown. Moreover, some inaccuracies in its performance may be attributable to errors in the computation of angular velocity, and not errors in its integration. Our main objective was to investigate the performance and capabilities of small ring-like attractor networks rather than to provide a detailed model of the fly HD circuit per se. As such, there are many differences between the fly circuit and the simple model we explore here, some of which may provide as-yet-undescribed mechanisms to overcome potential problems of discreteness. For example, a potential substrate for tuning local excitation may be the synaptic contacts that fly HD neurons make between themselves in different substructures of the CX^15,35^. Some of these and other fine-scale details of synaptic connectivity have not been incorporated into existing rate models^30,34^ or spiking neuron models^29,31,47^ of the circuit. In addition, these previous modeling efforts have focused on capturing the dynamics of the circuit without incorporating the biophysical properties of its neurons, and, in most cases, with only a subset of the excitatory and inhibitory cell types likely involved in generating the dynamics. Although the receptor and transmitter profiles of the relevant neurons are known^35^, further experiments are required to assess how intrinsic neuronal properties shape persistent population activity, as reported in the mammalian HD system^48^. Indeed, these intrinsic properties may account for the low drift we observed in the circuit (Fig. 1i) relative to that predicted by the model (Fig. 4d). Thus, while our work shows that small networks can, with appropriate tuning, implement continuous ring attractors, further experiments are needed to understand their cellular and synaptic implementation in real circuits.

Importantly, large ring attractor networks also suffer from the problem of fine-tuning, where noise in the connectivity—arising, for example, from heterogeneity in synaptic or cellular properties—can yield bumpy energy landscapes similar to those generated here (Fig. 2e). Several mechanisms have been proposed to combat this issue, including homeostatic synaptic scaling^49^ and synaptic facilitation^50^. These mechanisms might also be effective in the small networks studied here, where—in addition to fine-tuning the profile of the connectivity—the overall strength of local excitation must also be fine-tuned. Away from these optimal values, network dynamics are governed by unstable and stable linear regimes in which the population activity is pushed from or pulled toward discrete fixed points. We identified three properties of these regimes that govern network performance: the angular width of each regime, the locations of fixed points within each regime, and the speed at which the bump is pushed from or pulled toward each fixed point. Varying the strength of local excitation alters the balance between the regimes, such that improving performance in one regime worsens performance in the other. However, as the local excitation approaches an optimal value, the overall performance is dominated by the better-performing regime, which, in the same limit, becomes a ring attractor.

This analysis relied on characterizing the behavior of threshold-linear networks in terms of a separation between different linear dynamical regimes. This separation has recently been used to infer the underlying connectivity of biological networks^51^, and to design different connectivity motifs that generate distinct dynamical patterns, for example, to keep count or coarsely represent different positions^52,53^. Here, we showed how the precise tuning of interactions within a single connectivity motif shapes the properties of these linear regimes, and how these properties, in turn, affect performance. We found that certain regions of parameter space reduce drift and improve integration, and among these ‘good’ parameter regions, some are more robust than others. Specifically, we found that larger optimal values of local excitation, which generate narrower activity bumps, are more robust to variations in tuning and to additive noise, consistent with previous studies of noise robustness in attractor networks^3,46^.

Our results relied on specific assumptions about network connectivity and dynamics. We assumed local cosine-tuned excitation and broad uniform inhibition, but ring attractor manifolds can be generated with different hand-tuned^22,24,25,27,54^ or learned^55^ connectivity structures. Similarly, velocity integration can be performed in multiple ways, for example, using a network of two rings that receive differential velocity input^25^, or through two side rings that inherit heading activity from and project back to a center ring with velocity-dependent phase shifts^23^, as has been observed experimentally^30,37^. Our formulation approximates this second implementation in the limit that the side rings have fast neural time constants^24^. Finally, our choice of a threshold-linear response function enabled us to decompose the dynamics into distinct linear regimes^42,43^ that differentially affect performance, and it allowed us to analytically characterize the tuning precision required to achieve a desired level of performance. In such threshold-linear networks, this precision is limited to the tuned component of the connectivity; however, in networks with other nonlinearities, both the tuned and untuned components must be precisely chosen (Extended Data Fig. 5a). We expect such optimal tunings to exist more generally, provided that the energy of the system varies smoothly with the network tuning. In such cases, parameter-dependent changes in the stability of fixed points must be connected through optimal parameter tunings that locally flatten the energy as a function of orientation, as observed in Fig. 3f (Supplementary Note). In the absence of such tuning precision, small networks can fail to integrate velocity inputs and can drift in the absence of input. While such performance failures are known to arise in small attractor networks with differing connectivity structures and neural response functions^3,46^, it remains an open question how these different design features affect the relationship between tuning precision and performance more broadly.

While these results were motivated by and interpreted in the context of the small HD system of *Drosophila*, they immediately generalize to other scenarios. For example, the ring attractor network can be used to model place fields in circular environments, grid fields in one dimension, persistent-activity-mediated short-term memory of stimuli represented by angular variables^1^, and the preparation of motion toward targets on a circle^10^. Our results suggest that such representations could be accurately maintained using few neurons, thereby broadening the classes of computations that could be performed by small circuits. Moreover, these results could further generalize to higher-dimensional continuous variables, such as HD, place, and grid fields in two or three dimensions^9,17–19^ (see Extended Data Fig. 5b for proof-of-principle numerical results). More broadly, the ability to represent one continuous variable accurately using small numbers of neurons could more easily enable large systems to represent multiple continuous variables, such as the representation of many environments observed in the rodent hippocampus^5,20,21^.

## Methods

### Experimental setup

#### Fly preparation for imaging

We expressed the genetically encoded calcium indicator GCaMP7f (ref. ^56^) in EPG neurons by crossing GCaMP7f flies (w1118;;PBac[20XUAS-IVS-Syn21-op1-GCaMP7f-p10] in VK00005) to the EPG GAL4 driver line SS00096 (ref. ^57^). Flies (females, age 5–9 days, *n* = 10) were prepared for imaging as previously described^8,58^. Briefly, flies were anesthetized at 4 °C, their proboscis immobilized with wax to reduce brain movements, and their head/thorax fixed to a holder with a recording chamber using ultraviolet glue. To gain optical access to the brain, we removed a section of cuticle between the ocelli and antennae, along with the underlying fat and air sacs. Throughout the experiment, the head was submerged in saline containing NaCl (103 mM), KCl (3 mM), TES (5 mM), trehalose (8 mM), glucose (10 mM), NaHCO_3_ (26 mM), NaH_2_PO_4_ (1 mM), CaCl_2_ (2.5 mM) and MgCl_2_ (4 mM), with a pH of 7.3 and an osmolarity of 280 mOsm.

#### Two-photon calcium imaging

Calcium imaging was performed with a custom-built two-photon microscope controlled with ScanImage (version 2022, Vidrio Technologies)^59^. Excitation of GCaMP7f was generated with an infrared (920 nm), femtosecond-pulsed (pulse width ~110 fs) laser (Chameleon Ultra II, Coherent) with 15 mW of power, as measured after the objective (×60 Olympus LUMPlanFL/IR, 0.9 numerical aperture). Fast Z-stacks (eight planes with 6-μm spacing and three fly-back frames) were collected at 10 Hz by raster scanning (128 × 128 pixels, ~75 × 75 μm^2^) using an 8-kHz resonant-galvo system and piezo-controlled Z positioning. Focal planes were selected to cover the full extent of EPG processes in the EB. Emitted light was directed (primary dichroic: 735, secondary dichroic: 594), filtered (filter A: 680 SP, filter B: 514/44) and detected with a GaAsP photomultiplier tube (H10770PB-40, Hamamatsu).

#### Spherical treadmill system

Following dissection, flies were positioned on an air-supported polyurethane foam ball (8-mm diameter, 47 mg) under the two-photon microscope and allowed to walk. Rotations of the ball were tracked at 500 Hz, as described previously^58^. Behavioral data and imaging timestamps were recorded using WaveSurfer (version 0.947, http://wavesurfer.janelia.org/). For each fly, we collected five 20-min trials during which flies walked or stood in darkness.

### Data analysis

All data analysis was performed in MATLAB (version 2022a, MathWorks). Some analyses relied on functions from the Circular Statistics Toolbox (version 2012a)^60^. No statistical methods were used to predetermine sample sizes, but our sample sizes are similar to those reported in previous publications^8,30,61^. Flies were selected at random from their vials; however, as all data were collected from a single experimental condition (flies walking in darkness), no other randomization was performed. Data collection and analysis were not performed blind to the conditions of the experiments. We excluded any data collected beyond 100 min for consistency and to exclude a small number of flies whose behavior and/or imaging degraded in quality, a known limitation of fly-on-a-ball calcium imaging experiments.

#### Extracting bump orientation and strength

Each Z-stack was reduced to a single frame using a maximum-intensity projection technique. An ellipse was manually drawn around the perimeter of the EB and automatically segmented into 32 equal-area, wedge-shaped ROIs. The number of ROIs was chosen to be twice the number of anatomically defined EB wedges^62^. Activity within each ROI was averaged for each frame, producing 32 ROI time series. For each ROI time series, baseline fluorescence (*F*_0_) was defined as the average of the lowest 10% of samples. Δ*F*/*F* was computed as 100 × (*F* − *F*_0_)/(*F*_0_), where *F* is the instantaneous fluorescence from the raw ROI time series. These ROI time series were then smoothed with a third-order Savitzky–Golay filter over 11 frames as in previous studies^8,30^. We used the PVA as a measure of bump strength and orientation. PVA was computed by taking the circular mean of vectors whose angles were the ROI’s wedge positions and whose length was equal to the ROI’s Δ*F*/*F*. The magnitude of this mean resultant vector length was normalized to have a maximum possible length of 1.

#### Characterizing bump drift

To determine bump drift (Fig. 1h,i), we first identified periods when flies were standing still (defined as zero rotational and translational velocity), disregarding periods shorter than 300 ms. Drift was computed as the circular distance between bump orientations (PVA phase) at the beginning and end of these periods of standing. To determine whether the EPG bump drifted from its initial position to preferred discrete locations within the EB when the fly stood still, we compared the distributions of initial and final bump positions across 64 nonoverlapping bins from −π to π around the structure (Extended Data Fig. 1a,b). We used Watson’s *U*^2^ test^63,64^, a nonparametric two-sample test, for this comparison, implemented using MATLAB code from P. Mégevand (watsons_u2, https://github.com/pierremegevand/watsons_u2, 2017). We used 500 permutations to compute *P* values for this test; these *P* values, together with the test statistic *U*^2^, are reported in the caption of Extended Data Fig. 1b. Finally, we computed the distribution of drifts for periods between 300 ms and 2 s across 64 nonoverlapping binned initial positions from −π to π around the EB, and fit each fly’s drift distribution with sinusoidal functions of the form *A* × sin(*ω* × *ψ* + *θ*) + *C*, where *ω* ∈ {8, 16} is the frequency of the sinusoid, *ψ* is the initial bump position during the standing period, and *A*, *θ*, *C* are learned parameters for the amplitude, phase, and DC offset, respectively (Extended Data Fig. 1c,d). Frequencies of 8 and 16 Hz were chosen to match the number of computational units in the fly’s compass network, which, in a discrete network, would cause the bump to drift toward 8 (or 16) distinct bump positions (schematized in Fig. 1h, top). For each fly, we computed the *R*^2^ value between the drift, measured as a function of HD, and the sinusoidal fits (Extended Data Fig. 1c); these *R*^2^ values are reported in Extended Data Fig. 1d.

#### Characterizing bump velocity

To determine whether the EPG bump shows signs of nonlinear integration (Fig. 1j, top), we measured whether the bump moved faster or slower than expected as a function of bump position for both left and right turns (Fig. 1j, middle and bottom). We began by performing a linear regression (ordinary least squares) between the fly’s instantaneous angular velocity and the bump’s angular velocity (both sampled at 10 Hz) to account for fly-to-fly variability in the gain of angular integration, as observed in previous studies^8,30,61^. Linear fits were separately performed for left and right turns, and the residuals were taken as a measure of whether the bump was moving faster (or slower) than expected after accounting for each fly’s naive gain. Next, we binned data by bump position (64 nonoverlapping bins from −π to π) and computed the average residual bump velocity for each bin, producing the curves shown in the middle and bottom panels of Fig. 1j. Lastly, we fit each fly’s curve with sinusoidal functions of the form *A* × sin(*ω* × *ψ* + *θ*) + *C*, where *ω* ∈ {8, 16} is the frequency of the sinusoid, *ψ* is the bump position, and *A*, *θ*, *C* are learned parameters for the amplitude, phase, and DC offset, respectively (Extended Data Fig. 2). Frequencies of 8 and 16 Hz were chosen to match the number of computational units in the fly’s compass network, which, in a discrete network, would cause the bump to move faster or slower than expected at 8 (or 16) distinct bump positions (schematized in Fig. 1j, top). For each fly, we computed the *R*^2^ value between the residual bump velocity, measured as a function of HD, and the sinusoidal fits (Extended Data Fig. 2a); these *R*^2^ values are reported in Extended Data Fig. 2b.

We note that our fly-on-a-ball calcium imaging setup comes with potential challenges for evaluating the presence or extent of nonlinear integration, including slow GCaMP dynamics, altered proprioceptive feedback that the fly may experience while walking on a ball heavier than itself, head fixation that may prevent the fly from altering its head–body angle during turns, potential neural propagation delays involved in relaying and integrating the angular velocity signal, and measurement noise inherent to calcium imaging that could corrupt bump velocity estimation.

### Model overview

#### Network equations

We consider an effective single-ring network of *N* neurons (or, equivalently, of *N* computational units; see ‘Network equations’ in the Supplementary Note). Neurons are ordered according to their preferred heading *θ*_*j*_, which we take to be evenly spaced by Δ*θ* = 2π/*N* rad. Neurons are recurrently connected according to their preferred headings through a symmetric weight matrix ${W}_{jk}^{{\rm{\,sym}}}={J}_{\textrm{I}}+{J}_{\textrm{E}}\cos ({\theta }_{j}-{\theta }_{k})$, where *J*_E_ and *J*_I_ parametrize the strength of local excitation and uniform inhibition, respectively (note that *J*_E_ and *J*_I_ actually correspond to tuned and untuned components of the connectivity; for ease of language, we use local excitation and broad inhibition here and throughout). Neurons receive velocity input through an asymmetric, velocity-modulated weight matrix ${v}_{{\rm{in}}}{W}_{jk}^{{\rm{\,asym}}}={v}_{{\rm{in}}}\sin ({\theta }_{j}-{\theta }_{k})$; in the main text, we took *v*_in_ > 0. Each neuron *j* receives a constant feedforward input *c*_ff_ and a net input $1/N\,{\sum }_{k}({W}_{jk}^{{\rm{\,sym}}}+{v}_{{\rm{in}}}{W}_{jk}^{{\rm{\,asym}}}){r}_{k}$ from all other neurons in the network, where the firing rate *r*_*k*_ = *ϕ*(*h*_*k*_) is a nonlinear function of the total input activity *h*_*k*_. For all analyses shown in the main text, we took the nonlinear transfer function *ϕ*(⋅) to be rectified linear (that is, *ϕ*(⋅) = [⋅]_+_, but see also Extended Data Fig. 5 and ‘Robustness to changes in the transfer function and recurrent weights’ in the Methods). The dynamics of each neuron are given by the system of single-neuron equations in equation (1); we chose *τ* = 0.1 s and *c*_ff_ = 1.

By applying a discrete Fourier transform to the single-neuron equations, we can express this system of equations in terms of its Fourier modes. After initial transients, only the DC and first-order modes remain, and the resulting dynamical system reduces to a set of three equations that govern the dynamics of the orientation *ψ*, amplitude *a* relative to the average input activity, and width *w* of the bump (‘Order equations’ in the Supplementary Note); we will refer to these as the system of bump equations.

#### Stable parameter regime

The system of bump equations will generate a stable bump of activity for certain combinations of *J*_E_ and *J*_I_ (‘Fixed point analysis’ in the Supplementary Note and Extended Data Fig. 3a). For all analyses shown in the main text, we first selected a desired value of *J*_E_ > 2, and then selected a value of *J*_I_ such that it produced a bump of activity whose full amplitude *A* = *H*_0_ + *a* (where *H*_0_ is the average input activity) was at least approximately *A* = 0.2. To do so, we first uniformly sampled bump orientations *ψ* ∈ [0, 2π) and widths *w* ∈ [2π/*N*, 2(*N* − 1)π/*N*), and we used these to calculate the contour *J*_E_*f*_even_(*w*, *ψ*) = 1 using MATLAB’s ‘contourc.m’, where *f*_even_(*w*, *ψ*) is given by equation (S19) in the Supplementary Note (see also equation (S30) in the Supplementary Note and Extended Data Fig. 3c). This gave us values $(w,\psi )\in {C}_{{J}_{\textrm{E}}}=\{(w,\psi )\,| \,{J}_{\textrm{E}}{f}_{{\rm{even}}}(w,\psi )=1\}$ that satisfy the contour equation. We then used these values of *w* and *ψ* to determine an upper bound on *J*_I_ given by

8 $${J}_{\textrm{I}}^{{\,\rm{bound}}}=\mathop{\min }\limits_{(w,\psi )\in {C}_{{J}_{\textrm{E}}}}\frac{-\cos (w/2)}{{f}_{0}(w,\psi )},$$

where *f*_0_(*w*, *ψ*) is given by equation (S18) (see also equation (S32)) in the Supplementary Note. We then used these same values of *w* and *ψ* to determine a value for *J*_I_, given by

9 $${J}_{\textrm{I}}=\mathop{\min }\limits_{(w,\psi )\in {C}_{{J}_{\textrm{E}}}}\frac{({c}_{\textrm{ff}}/A-1)\cos (w/2)-{c}_{\textrm{ff}}/A}{{f}_{0}(w,\psi )},$$

and verified that ${J}_{\textrm{I}} < {J}_{\textrm{I}}^{{\,\rm{bound}}}$. Plugging *A* = 0.2 into equation (9) resulted in a bump of activity whose minimum full amplitude was approximately *A* = 0.2.

### Model analytics

#### Stationary solutions

To determine the configurations to which the system evolves in the absence of velocity input, we characterized the stationary solutions of the system of bump equations (‘Fixed point analysis’ in the Supplementary Note). This allowed us to determine relationships between the bump orientation, relative amplitude, and width that would persistently maintain a stable bump of activity (Extended Data Fig. 3b,c). For a network of *N* neurons that receive no velocity input, most parameter settings will yield two sets of *N* fixed points each—one set will be stable, and the other will be unstable. For a given value of *J*_E_, one set will be aligned with the preferred headings {*θ*_*j*_}, and the other set will be aligned precisely between the preferred headings; the second and fourth columns of Fig. 2e highlight examples for which the unstable (second column) and stable (fourth column) sets of fixed points are aligned with the preferred headings. The value of *J*_E_ and the parity of *N* (whether the network consists of an even or odd number of neurons) together specify which of these two configurations the network will adopt. When *N* is even and ${J}_{\textrm{E}} < {J}_{{\textrm{E}},N-2}^{* }$ (denoting bumps supported by *N* − 1 and *N* − 2 neurons), the set of fixed points aligned with the preferred headings will be unstable. When *N* is odd, the reverse will be true: for ${J}_{\textrm{E}} < {J}_{{\textrm{E}},N-2}^{* }$, the set of fixed points aligned with the preferred headings will be stable. For a given network size *N*, as *J*_E_ passes through an optimal value ${J}_{\textrm{E}}^{* }$, this stability switches (Extended Data Fig. 3d,g). At each of these fixed points, the widths of the stable and unstable bump configurations are determined solely by *J*_E_, whereas their relative amplitudes depend on both *J*_E_ and *J*_I_.

#### Energy landscape

We derived an energy landscape *E*(*a*, *w*, *ψ*; *J*_E_, *J*_I_) for the system of bump equations in the absence of velocity input^40,41^ (‘Energy landscape’ in the Supplementary Note). This function describes the stable configurations to which the system will evolve in the absence of input.

To minimize the curvature of the energy landscape, we first determined the 3 × 3 Hessian matrix of the second derivatives of the energy *E* with respect to *a*, *w*, and *ψ*. When evaluated at the orientations *ψ*^s^ of the stable fixed points (see the previous subsection), we found that the Hessian reduced to a block diagonal matrix, with a single eigenvector along *ψ* whose eigenvalue is given by

10 $$\frac{{\partial }^{2}E}{\partial {\psi }^{2}}\propto 1-\frac{{J}_{\textrm{E}}}{N}\sum _{k\in {K}_{{\rm{act}}}}{\sin }^{2}({\theta }_{k}-{\psi }^{\textrm{s}}),$$

where *K*_act_ denotes the set of indices of the neurons that actively maintain the bump. This eigenvalue quantifies the degree of local curvature of the energy as a function of bump orientation *ψ*. For a system of size *N*, there are *N* − 3 values of local excitation *J*_E_ for which this eigenvalue goes to zero, and thus for which the energy landscape is locally flat as a function of *ψ*. These correspond to bump configurations for which the bump is maintained by *N*_act_ ∈ [2, *N* − 2] active neurons:

11 $$\frac{1}{{J}_{{\textrm{E}},{N}_{{\rm{act}}}}^{* }}=\frac{1}{4}+\frac{1}{2N}\left(\tilde{n}+\frac{\sin (2\uppi \tilde{n}/N)}{\sin (2\uppi /N)}\right);\,\tilde{n}={N}_{{\rm{act}}}-\frac{N}{2}.$$

We found that these values of local excitation, which are shown in Fig. 2d, also ensure that the energy landscape is flat for all bump orientations (as shown in Fig. 2e; also see Extended Data Fig. 4).

#### Leading eigenvalues of active submatrices

In the absence of velocity input, the bump dynamics are governed by the leading eigenvalue *λ* of a submatrix of the connectivity (−*I* + *W*^sym^/*N*)/*τ*; this eigenvalue determines the rate at which the bump will drift in the absence of input. When the local excitation *J*_E_ is optimally tuned (that is, ${J}_{\textrm{E}}={J}_{{\textrm{E}},{N}_{{\rm{act}}}}^{* }$), the bump of activity will be maintained by a fixed number of active neurons *N*_act_ ∈ [2, …, *N* − 2]. For each distinct value of *N*_act_, there is thus a distinct *N*_act_ × *N*_act_ submatrix of the connectivity whose single leading eigenvalue determines the drift dynamics. Away from these optimal values of local excitation, the bump of activity will be maintained by either *n* or *n* + 1 active neurons (see equation (S50) in the Supplementary Note). The drift dynamics are then governed by the leading eigenvalues of the corresponding *n* × *n* and (*n* + 1) × (*n* + 1) active submatrices.

To determine these dynamics, we analytically determined the rates of bump drift in the stable and unstable regimes, which are given in equation (2) (see ‘Performance of non-optimal solutions: Dynamics in the absence of input velocity’, and, in particular, equations (S54) and (S56) in the Supplementary Note). We then compared these analytically derived drift rates to the leading eigenvalues that we computed numerically by directly diagonalizing active submatrices of the connectivity (using the MATLAB function ‘eig.m’); this comparison is shown in Extended Data Fig. 6.

#### Widths of stable and unstable regimes

In the absence of input, the widths of the stable and unstable regimes can be determined analytically by finding the orientation at which the bump transitions from unstable to stable dynamics as it drifts away from an unstable fixed point. This reduces to matching two exponential equations that govern the dynamics of the bump orientation in the two regimes (with drift rates *λ*_u_ and *λ*_s_, respectively), and that must tend toward the orientations of the unstable and stable fixed points as *t* → −∞ and *t* → +∞, respectively. The resulting widths of each regime are given by equation (3) and shown in Fig. 3d and Extended Data Fig. 8b, and they are centered on the orientations of the stable and unstable fixed points in the absence of input. Given a stable fixed point at *ψ* = *ψ*^s^ and an unstable fixed point at *ψ* = *ψ*^u^ = *ψ*^s^ + π/*N*, the resulting equation for the bump can then be written as (see equations (S61) and (S62) in the Supplementary Note):

12 $$\psi (t)=\left\{\begin{array}{l}{\psi }^{\textrm{u}}+({\psi }_{0}-{\psi }^{\textrm{u}})\exp ({\lambda }_{\textrm{u}}t)\,\,;\quad 0 < t < {t}_{\Delta n}\quad \,\text{unstable regime}\,\quad \\ {\psi }^{\textrm{s}}+\frac{\Delta {\theta }_{\textrm{s}}}{2}\exp (-| {\lambda }_{\textrm{s}}| (t-{t}_{\Delta n}))\,;\quad t > {t}_{\Delta n}\quad \,\text{stable regime}\,\quad \end{array}\right.,$$

where *ψ*^s^ + Δ*θ*_s_/2 < *ψ*_0_ < *ψ*^u^ is the initial orientation of the bump, and *t*_Δ*n*_ = (1/*λ*_u_) log(Δ*θ*_u_/(2(*ψ*^u^ − *ψ*_0_))) is the time when the bump orientation crosses from the unstable regime into the stable regime. See ‘Performance of non-optimal solutions: Dynamics in the absence of input velocity’ in the Supplementary Note for more details.

#### Drift in the absence of input

To measure the net bump drift, we analytically computed the time *τ*_d_ that it takes for the bump to drift from within *ε*_u_ of an unstable fixed point to within *ε*_s_ of a stable one. We chose *ε*_u_ = Δ*θ*_u_/2e and *ε*_s_ = Δ*θ*_s_/2e, such that the bump covered an angular distance of Δ*ψ*_d_ = (1 − 1/e)Δ*θ*/2 in the time *τ*_d_. We then measured the net drift speed as Δ*ψ*_d_/*τ*_d_ (see equations (S68)–(S71) in the Supplementary Note).

#### Small velocity approximation

In the presence of velocity input, the bump dynamics will be governed by the leading eigenvalue *λ* of a submatrix of the full connectivity (−*I* + (*W*^sym^ + *v*_in_*W*^asym^)/*N*)/*τ*. The asymmetric component of this connectivity is modulated by the input velocity *v*_in_, and introduces a velocity-dependent correction to the eigenvalue *λ*_0_ of the symmetric connectivity (−*I* + *W*^sym^/*N*)/*τ* (Extended Data Fig. 7):

13 $$\lambda \approxeq {\lambda }_{0}+f(\,{J}_{\textrm{E}}){v}_{{\rm{in}}}^{2}+{\mathcal{O}}({v}_{{\rm{in}}}^{3}).$$

For sufficiently small input velocities, we can approximate the leading eigenvalues *λ*_u_ and *λ*_s_, and thus the corresponding widths of the unstable and stable regimes, as being equal to their values in the absence of velocity input (see ‘Leading eigenvalues of active submatrices’ and ‘Widths of stable and unstable regimes’ in the Methods). All analytic results shown in Fig. 3i–l were generated under this assumption. This approximation breaks down as the input velocity increases, and it breaks down more quickly for smaller values of local excitation (as shown in Fig. 3l; see also Extended Data Fig. 7a).

#### Locations of fixed points in a velocity-driven regime

Although we can approximate the rates and width of the stable and unstable regimes as remaining unchanged for a sufficiently small velocity input, we cannot make the same approximation for the orientations of stable and unstable fixed points. Therefore, we will treat the stable and unstable fixed-point orientations as functions of *v*_in_: *ψ*^s^ = *ψ*^s^(*v*_in_), *ψ*^u^ = *ψ*^u^(*v*_in_), respectively. The orientation of the stable and unstable fixed points found in the absence of velocity input will then be given by *ψ*^s^(0) and *ψ*^u^(0), respectively. To determine how the orientations of these fixed points shift with velocity, we repeated the analyses described in ‘Widths of stable and unstable regimes’ in the Methods, but with a different set of initial conditions (see ‘Performance of non-optimal solutions: Dynamics in the presence of small input velocity’ in the Supplementary Note for details). Given a bump that begins at a stable fixed point *ψ* = *ψ*^s^(0) in the absence of input, and given an initial velocity *v*_in_, the bump will be driven to a new stable fixed point at an orientation *ψ*^s^(*v*_in_) = *ψ*^s^(0) + *v*_in_/|*λ*_s_| as *t* → ∞. In the limit that *t* → −∞, the bump will be driven to (and hence, in forward time, away from) an unstable fixed point at an orientation *ψ*^u^(*v*_in_) = *ψ*^u^(0) − *v*_in_/*λ*_u_. Over an interval *ψ* ∈ [*ψ*^s^(0) − Δ*θ*_s_/2, *ψ*^u^(0) + Δ*θ*_u_/2], the resulting equation for the bump can be written as (see equations (S78) and (S79) in the Supplementary Note):

14 $$\begin{array}{l}\psi (t)=\left\{\!\begin{array}{l}{\psi }^{\textrm{s}}(0)+\frac{{v}_{{\rm{in}}}}{| {\lambda }_{\textrm{s}}| }\left(1-\exp (-| {\lambda }_{\textrm{s}}| t )\right);\qquad \qquad0 < t < {t}_{\textrm{c}}\;\,\text{stable regime}\\ {\psi }^{\textrm{u}}(0)-\frac{{v}_{{\rm{in}}}}{{\lambda }_{\textrm{u}}}+\left(\frac{{v}_{{\rm{in}}}}{{\lambda }_{\textrm{u}}}-\frac{1}{2}(\Delta \theta -\Delta {\theta }_{\textrm{s}})\right)\\\qquad\;\;\exp ({\lambda }_{\textrm{u}}(t-{t}_{\textrm{c}}));\;\qquad\qquad\qquad \quad\;\;{t}> {t}_{\textrm{c}}\qquad\;\text{unstable regime} \end{array}\right.,\end{array}$$

where *t*_c_ = (1/|*λ*_s_|) log(1/(1 − Δ*θ*_s_|*λ*_s_|/2*v*_in_)) is the time when the bump orientation crosses from the stable regime into the unstable regime.

At the threshold velocity given in equation (5), the two fixed points will meet at the boundary between regimes; this is the minimum velocity needed for the bump to move continuously. Below this velocity, the bump will be driven away from the unstable fixed point in the unstable regime, and toward a stable fixed point in the stable regime. Above this velocity, the stable and unstable fixed points will still drive the bump dynamics, but their orientations will move outside of their respective regimes. The minimum and maximum bump velocities, ${{\nu }_{\min }}$ and ${{\nu }_{\max }}$ (given by equation (6)), can be computed analytically from equation (14) by evaluating the time derivative of *ψ*(*t*) at the boundary from the stable to the unstable regime, and vice versa. We used these minimum and maximum velocities to define the linearity of integration as ${{\nu }_{\min }}/{{\nu }_{\max }}$. See ‘Performance of non-optimal solutions: Dynamics in the presence of small input velocity’ in the Supplementary Note for details.

#### Simplified energy landscape

Having described each linear subsystem in terms of (1) the orientations of the fixed points, (2) the rate at which the bump drifts toward or away from these fixed points, and (3) the angular regime governed by each fixed point, we used these three properties to construct a simplified landscape that describes the energy of different bump orientations. Given a linear system, an energy function can be chosen to be quadratic^65^; we thus choose *E*_u,s_(*ψ*) = *α*_u,s_*ψ*^2^, where *α*_s_ > 0 for the stable subsystem, and *α*_u_ < 0 for the unstable subsystem. To select the appropriate values of *α*_u,s_, we require that the energy function has extrema at the orientations of the stable and unstable fixed points *ψ*^s^(*v*_in_) and *ψ*^u^(*v*_in_), and that the energy transitions smoothly between the stable and unstable regimes; this yields

15 $$E(\psi )=\left\{\begin{array}{l}-{\lambda }_{\textrm{u}}{(\psi -{\psi }^{\textrm{u}}({v}_{{\rm{in}}}))}^{2}+C({v}_{{\rm{in}}})\quad \,\text{unstable regime}\,\quad \\ -{\lambda }_{\textrm{s}}{(\psi -{\psi }^{\textrm{s}}({v}_{{\rm{in}}}))}^{2}\quad \quad \quad \quad \quad \,\text{stable regime}\,\quad \end{array}\right.,$$

where $C({v}_{{\rm{in}}})=\Delta \theta \left({\lambda }_{\textrm{u}}\Delta {\theta }_{\textrm{u}}/4-{v}_{{\rm{in}}}+{v}_{{\rm{in}}}^{2}/({\lambda }_{\textrm{u}}\Delta {\theta }_{\textrm{u}})\right)$, and where *α*_u_ = −*λ*_u_ < 0 and *α*_s_ = −*λ*_s_ > 0 as required. When moving around the ring, each successive pair of stable and unstable regimes will be governed by an energy landscape of this form but with a vertical shift, such that *E*(*ψ* ± *n*Δ*θ*) = *E*(*ψ*) ∓ 2*nv*_in_Δ*θ*. See ‘Simplified energy’ in the Supplementary Note for more details.

#### Tolerance in tuning

To determine how precisely the local excitation must be tuned to achieve a criterion level of performance, we first computed the derivative of each performance measure as a function of local excitation, evaluated at an optimal value; we denote this ${m}_{P}(\,{J}_{\textrm{E}}^{* })$ (see equations (S96)–(S99) in the Supplementary Note). This slope gives us a local linear estimate of how quickly the performance degrades away from an optimal value of local excitation. Because each performance measure can be expressed as a function of the net drift speed |*λ*_d_|, computing this slope reduced to computing $\partial | {\lambda }_{\textrm{d}}| /\partial {J}_{\textrm{E}}{| }_{{J}_{\textrm{E}}^{* }}$. Given a criterion for the system to be within ${\varepsilon }_{P}^{{\rm{tol}}}$ of optimal performance for a performance measure *P*, the tolerance about a given optimal value ${J}_{\textrm{E}}^{* }$ can then be computed as $\left.{\mathrm{tol}}_{P}(\,{J}_{\textrm{E}}^{* })\ge 2{\varepsilon }_{P}^{{\rm{tol}}}\right/| {m}_{P}(\,{J}_{\textrm{E}}^{* })|$ (where ≥ indicates that this is a lower bound on the tolerance, as the linear slope will overestimate the rate of degradation of performance; see equation (S113) in the Supplementary Note).

To determine the volume of parameter space that can meet this desired performance, we summed the tolerance across all optimal values of local excitation for a given network size *N* (see equation (S120) in the Supplementary Note). We then approximated this sum by its largest value, which reduces to

16 $$V(N)\ge {c}_{P}\frac{{N}^{2}}{1-\cos (2\uppi /N)}.$$

See ‘Degradation of performance as a function of local excitation’ in the Supplementary Note for more details.

### Model simulations

#### Overview

All simulations that we performed used MATLAB’s ODE solver ‘ode45.m’ with an integration timestep of Δ*t* = 0.01 s. We first initialized the network to generate a bump of activity at a given orientation *ψ*. Using this as the initial condition for the network, we then simulated the single-neuron dynamics in equation (1), and we performed a discrete Fourier transform using MATLAB’s ‘fft.m’ function to extract the bump dynamics as a function of the single-neuron dynamics (see equation (S16) in the Supplementary Note). When simulating angular velocity integration, we first determined the velocity scaling that would generate a comparable rate of bump movement for a given (constant) velocity input (see ‘Velocity-driven dynamics’ in the Methods). We then simulated the network dynamics in response to this scaled input.

#### Parameter choices

All results shown in Figs. 2 and 3 were generated using networks of size *N* = 6. When illustrating network properties for different values of local excitation, we used the following values of *J*_E_ (evenly spaced in 1/*J*_E_): *J*_E_ = [12, 6, 4, 3, 2.4] (Fig. 2e–h); *J*_E_ = [3.89, 3.6, 3.3, 3, 2.77, 2.57, 2.44] (Fig. 3f,j); *J*_E_ = [3.6, 3, 2.57, 2.44] (Fig. 3g,k); 17 evenly spaced values of 1/*J*_E_ between 1/3.86 and 1/2.45 (Fig. 3h,l). When simulating network dynamics in the presence of velocity input, we used the following values of velocity input *v*_in_: ten evenly spaced velocity values between 0.2 and 2.0 rad s^−1^ (Fig. 2f); ten evenly spaced values between 0.1 and 1.0 rad s^−1^ (Fig. 3k); five evenly spaced values between 0.8 and 1.6 rad s^−1^ (Fig. 3l). In all cases, we scaled the velocity input as described below (see ‘Velocity-driven dynamics’ in the Methods).

#### Drift in the absence of input

For simulations of bump drift, we simulated the network with the velocity input set to zero. To illustrate drift trajectories for different values of *J*_E_ (as shown in the bottom row of Fig. 2f and in Fig. 3g), we initialized the bump at six evenly spaced orientations between (and including) 0 and π/*N*, and we simulated the evolution of the bump for 3 s. We repeated this for repeating angular units between 0 and 2π.

#### Measuring net drift speed

To measure the net drift speed (as described in ‘Drift in the absence of input’ in ‘Model analytics’ in the Methods), we initialized the bump at an orientation *ψ*^u^ − *ε*_u_ (where *ψ*^u^ is the orientation of an unstable fixed point; for the values of *J*_E_ used in Fig. 3, *ψ*^u^ = π/*N*; see ‘Parameter choices’ in the Methods). We then simulated the network dynamics until the bump reached an orientation *ε*_s_. We set *ε*_u_ = Δ*θ*_u_/2e and *ε*_s_ = Δ*θ*_s_/2e, where Δ*θ*_u,s_ were computed as described in ‘Widths of stable and unstable regimes’ in the Methods. We used the time it took for the bump to reach this orientation as the measure of the net drift timescale *τ*_d_, and we used Δ*ψ*_d_/*τ*_d_ as a measure of net drift speed, where Δ*ψ*_d_ = (1 − 1/e)Δ*θ*/2 is the angular distance traveled by the bump in the time *τ*_d_. Fig. 3h compares the net drift speed from simulations to that obtained analytically for different values of *J*_E_.

#### Velocity-driven dynamics

For simulations of angular velocity integration, we injected a constant velocity input throughout the simulation. To permit a comparison to analytic predictions, we scaled the input velocity such that the rate of movement of the bump matched the input velocity at an input of *v*_in_ = 50 rad s^−1^. To this end, we determined the best-fitting linear trajectory that minimized the absolute deviation from the bump trajectory over a time window of *t* = 6 s, and we used the slope of this linear trajectory to scale all other input velocities injected into the network. We performed this scaling separately for each set of network parameters (that is, for each choice of (*J*_E_, *J*_I_)). All velocity values described in simulations were scaled in this way.

#### Measuring threshold velocity

To measure the threshold velocity required to move the bump continuously (as shown in Fig. 3l), we first analytically computed the threshold velocity as described in ‘Locations of fixed points in a velocity-driven regime’ in the Methods. We then chose 50 evenly spaced input velocity values between (and including) *v*_thresh_ − 0.05 rad s^−1^ and *v*_thresh_ + 0.05 rad s^−1^. We initialized the bump at the orientation of a stable fixed point (here, at *ψ*^s^ = 0), and we then simulated the network dynamics in response to each velocity individually. We determined the minimum of these velocities that would move the bump beyond an orientation of π/*N* within a time interval of 10 s. Fig. 3l compares this simulated value to the value obtained analytically.

#### Measuring the linearity of integration

To measure the linearity of integration from simulations, we simulated the bump trajectory for different constant input velocities (as described above in ‘Overview’). For each input velocity, we determined the time *t*_c_ when the bump orientation *ψ* crossed from the stable into the unstable regime or vice versa; these times were used to compute the minimum and maximum velocities, respectively (note that we used the analytically derived boundaries between regimes to determine these crossing times; see ‘Widths of stable and unstable regimes’ in the Methods). We then determined the bump velocity as *ν* = (*ψ*(*t*_c_ + Δ*t*) − *ψ*(*t*_c_ − Δ*t*))/2Δ*t*, where Δ*t* = 0.1 s is the integration timestep used in all simulations. Fig. 3l compares this simulated value to the value derived analytically (see ‘Locations of fixed points in a velocity-driven regime’ in the Methods).

#### Robustness to variations in parameter tuning

To summarize performance as a function of network size (shown in Fig. 4a), we analytically computed the net drift speed (as described in ‘Drift in the absence of input’ in ‘Model analytics’ in the Methods) as a function of local excitation in the range ${J}_{\textrm{E}}\in [\,{J}_{{\textrm{E}},N-2}^{* },{J}_{{\textrm{E}},2}^{* }]$ (that is, between the minimum and maximum optimal values of local excitation, maintained by *N*_act_ = *N* − 2 and *N*_act_ = 2 active neurons, respectively). For each optimal value of local excitation, we numerically estimated the tolerance as the range of local excitation values about an optimum for which the net drift speed would be consistently below a fixed performance threshold (we used a threshold value of 0.001 rad s^−1^). We considered only those values of local excitation above the minimum optimal value or below the maximum optimal value to estimate this tolerance; thus, to estimate the tolerance about the minimum and maximum optimal values, we measured the tolerance in only one direction (${J}_{\textrm{E}}\le {J}_{\textrm{E},2}^{* }$ or ${J}_{\textrm{E}}\ge {J}_{{\textrm{E}},N-2}^{* }$), and we doubled this value to use as our estimate. We then compared these tolerance estimates to the analytic lower bound given in equation (7), as shown in Fig. 4b,c (also see equations (S113)–(S119) in the Supplementary Note). Finally, we summed these tolerance values (computed numerically or analytically) for each network size *N* to estimate the net volume of parameter space that meets this threshold level of performance, as shown in Extended Data Fig. 9a.

#### Robustness to noise

To measure noise robustness, we added independent Gaussian noise with variance *σ*^2^ to each neuron in our optimal networks, and we simulated network dynamics in the absence of velocity input. We ran 10,000 simulations in which we tracked the orientation of the bump over a total time of 20 s, and we used this to measure the variance of the difference between the initial and final bump positions over time: 〈(*ψ*(*t*) − *ψ*_0_)^2^〉. For short timescales, the dynamics of this quantity are affected by the finite integration timescale *τ*; at longer timescales, this quantity follows a diffusion equation with diffusion constant *D*: $\langle {(\psi (t)-{\psi }_{0})}^{2}\rangle ={\sigma }_{0}^{2}+2Dt$. We used the bump trajectories for *t* > 10 s to fit a value for 2*D*, as shown in Fig. 4d, and we took 1/2*D* as a measure of noise robustness. Figure 4e,f measures this robustness for optimally tuned networks of varying ${J}_{\textrm{E}}^{* }$ and *N*, and for varying noise levels: *σ*^2^ = (*A*/6)^2^ × [1, 4, 9, 16, 25], where *A* = 0.2 is the bump amplitude. To extract the dependence on *N* and *σ*^2^, as shown in Fig. 4f, we found the best-fitting coefficients *a*, *b* for the linear relationship 2*D* = (*aN* + *b*)/*σ*^2^ (see Extended Data Fig. 9b for a visualization of these coefficients).

#### Robustness to changes in the transfer function and recurrent weights

We examined the robustness of the continuous attractor regime to changes in the number of Fourier modes of the recurrent connections in *W*^sym^, the neuron input–output relationship *ϕ*, and an increase in the dimensionality of the attractor. To this aim, we numerically solved the dynamics of equation (1) with *v*_in_ = 0 in two different scenarios. First, we used (1) a von Mises connectivity profile with concentration parameter *κ* for the recurrent weights ${W}_{jk}^{{\rm{\,sym}}}={J}_{\textrm{I}}+{J}_{\textrm{E}}\exp (\kappa \cos ({\theta }_{j}-{\theta }_{k}))/(2\uppi {I}_{0}(\kappa ))$, where *I*_0_(*κ*) is the modified Bessel function of order 0; (2) a smooth nonlinear transfer function, *ϕ*(*x*) = log(1 + e^*x*^). We numerically solved the dynamics of a network with *N* = 8 units and *J*_I_ = −30, with cosine-shaped initial conditions centered at 50 uniformly spaced orientations on the ring (Extended Data Fig. 5a). We evaluated the dispersion (circular variance) between the initial and final orientations on the ring for different values of *J*_E_ after numerically solving the dynamics for a total time of 500*τ*, where *τ* is the single-neuron time constant. We observed the presence of optimal values of *J*_E_ (Extended Data Fig. 5a, red), where the network behaved like a continuous attractor, as opposed to other values of *J*_E_ (Extended Data Fig. 5a, purple, blue) where the discreteness of the solution was evident. The specific values of optimal excitation depend on both the value of *J*_I_ (Extended Data Fig. 5a, empty circles), and on the strength of constant feedforward input *c*_ff_.

We next examined the dynamics in equation (1) with a recurrent weight profile storing a two-dimensional toroidal attractor with *N* = 16 neurons, ${W}_{jk}^{{\rm{\,sym}}}={J}_{\textrm{I}}+\frac{{J}_{\textrm{E}}}{2}(\cos ({\theta }_{j}^{1}-{\theta }_{k}^{1})+\cos ({\theta }_{j}^{2}-{\theta }_{k}^{2}))$, *J*_I_ = −20, where the preferred orientations $({\theta }_{i}^{1},{\theta }_{i}^{2})$ of the units were uniformly spaced on the torus (Extended Data Fig. 5b). We similarly observed the presence of an optimal value of *J*_E_ for which the dispersion between subthreshold bumps initialized at 100 different orientations on the torus and the final orientations were close to 0.

### Reporting summary

Further information on research design is available in the Nature Portfolio Reporting Summary linked to this article.

## Online content

Any methods, additional references, Nature Portfolio reporting summaries, source data, extended data, supplementary information, acknowledgements, peer review information; details of author contributions and competing interests; and statements of data and code availability are available at 10.1038/s41593-024-01766-5.

## Supplementary information

Supplementary Information Supplementary Note.

Reporting Summary

## Data Availability

All data collected for this study are freely available via figshare at 10.25378/janelia.26169355 (ref. ^66^).

## Code availability

All custom code written for this study is freely available via Zenodo at 10.5281/zenodo.12789923 (ref. ^67^) and is maintained on GitHub at https://github.com/HermundstadLab/DiscreteRingAttractor.
