# Supplementary material for: Maintaining and updating accurate internal representations of continuous variables with a handful of neurons
Source: Nat Neurosci. 2024 Oct 3;27(11):18. doi: 10.1038/s41593-024-01766-5 (PMC11537979; doi:10.1038/s41593-024-01766-5)
Supplement: Supplementary file 1 — Supplementary Note. [file 41593_2024_1766_MOESM1_ESM.pdf]

nature neuroscience

Supplementary information

https://doi.org/10.1038/s41593-024-01766-5

Maintaining and updating accurate internal representations of continuous variables with a handful of neurons

In the format provided by the authors and unedited

SUPPLEMENTAL INFORMATION: THEORY & ANALYTICS

Terminology and Assumptions

Ring attractors have become a rather ubiquitous concept in neuroscience, often invoked as a solution to how neural systems perform computations that involve representations of continuous variables (e.g., those involved in working memory [1–3] and various types of integration [4–16]). As such, the terms "ring attractor" and "ring attractor network" have often been used more colloquially to describe systems that are thought to perform such computations (e.g., head direction systems). Here we seek to clarify the technical definitions of these terms as they relate to the results and discussion presented in the main text.

A *point attractor* (also referred to as a *discrete attractor*) is an isolated stable fixed point to which the network dynamics will converge when sufficiently close. In technical terms, it is a locally asymptotically stable state of the network dynamics. A *continuous attractor* is composed of a continuum of *marginally stable* fixed points, so termed because perturbations along this continuum will be maintained, whereas perturbations off of this continuum will converge back to a state along the continuum. A *ring attractor* is a continuous attractor for which the continuum of marginally stable fixed points is topologically arranged along a ring in state space. If a network has an attractor of type X, it is often referred to as an X *attractor network* (e.g., a *ring attractor network*). Networks that have attractors are broadly referred to as *attractor networks*.

Attractors that closely approximate a continuous attractor have been referred to as *quasi-continuous attractors* [4, 17]. These attractors are not formally continuous, in the sense that they do not possess a continuum of marginally stable fixed points. Rather, the continuum is usually approximated by a large number of closely-spaced point attractors. In the literature, both continuous and quasi-continuous attractors are often colloquially referred to as continuous attractors [17]. There is no clear definition of what constitutes a sufficiently "good" approximation of a continuous attractor to be deemed quasi-continuous. This has resulted in rather loose usage of the terms "continuous attractor" and "ring attractor". To avoid this ambiguity, we reserve the term "continuous attractor" for formally continuous attractors, and we reserve the term "ring attractor" for the special class of continuous attractor whose states lie along a ring. Note that our analysis of suboptimally-tuned networks (see *Results* | *Variations in tuning degrade network performance* in the main text) show how network size impacts different approximations to a ring attractor, thereby providing one potential framework for quantifying the "goodness" of such approximations. This analysis is valid for well-separated point attractors along a ring (when N is small) and for *quasi-ring attractors* (when N is large).

Another common point of confusion arises when distinguishing the neural representation of a variable from the underlying topology of the attractor along which the representation evolves. For example, in this work we consider the neural representation of an angular variable. This variable can be encoded by a ring attractor, where the continuum of marginally stable states along the ring are used to represent a continuum of angles, or by a discrete set of point attractors arranged along a ring, where each point attractor is used to stably represent a single angle. In both cases, the variable is circular and continuous, but the attractor is either continuous and circular (in the former case), or discrete and circular in arrangement (in the latter case). In the latter case, intermediate angles can be transiently represented by the network, but they cannot stably persist in the absence of input. Thus, attractor networks are capable of representing variables that do not match the topology of the attractor, but only those representations that correspond to the attractors can be held in memory and persist in the absence of input. Moreover, the topology of the attractor need not be mapped to the topology of the variable being represented; for example, one can map states along a ring attractor to any bounded interval, and can thus be used to represent bounded aperiodic continuous variables [18].

Finally, we distinguish between properties of a *network*, and properties of the *attractor* that the network generates. We refer to networks with finite size ($N < \infty$) as *discrete networks*, and networks with infinite size ($N \to \infty$) as *continuous networks*. With these definitions, a continuous network need not generate a continuous attractor, and a discrete network need not generate discrete (or point) attractors. This distinction becomes important here, because much of the theoretical analysis of (continuous) ring attractors has been done in the context of continuous networks [12, 19–21]. In these studies, it is typically assumed that N is large, and hence taking $N \to \infty$ provides a good approximation of the network dynamics. This leads to a continuous symmetry in the network, and hence, given a fixed point solution u, any translation of u will also be a fixed point solution. This family of translations defines the

marginally-stable states of the ring attractor. By contrast, a network of size $N < \infty$ has only an N-fold symmetry, suggesting that, given a stable fixed point solution u, there will be at most N stable fixed points corresponding to the N translations of u that lead to a uniform partitioning of orientation space. This intuition is consistent with the perspectives and findings laid out in previous studies, which have claimed that nonlinear networks of finite size will necessarily have a finite number of point attractors [22, 23] that in turn result in discretization errors in the absence of input and failures to integrate small inputs [17]. Consistent with these claims, large networks have been shown to result in better angular velocity integration, even under deterministic conditions [24]. Taken together, these results suggest that generating a proper ring attractor requires $N \to \infty$; we work under this *a priori* assumption based on the following observations:

1. To our knowledge, no existing study demonstrates that small networks can generate ring attractors.
2. The majority of existing theoretical and computational work on ring attractors either uses large N (e.g., [1, 3, 6, 24]) or takes $N \to \infty$ (e.g., [12, 19–21]).
3. Existing simulations in small networks show clear signatures of discreteness; these include having a small number of well-separated stable states (see Fig. 10 in [22] and Fig. 2E in [25]) and the inability to integrate small inputs (see Fig. 10E in [26]).

To our knowledge, our study is the first to show that a proper ring attractor can be generated in a discrete (and small) network.

Network Equations

We consider a simplified system with a triple ring structure, with each ring composed of the same number of computational units, N. Here, computational units can be single neurons, or they can by groups of neurons with the same tuning and connectivity. In what follows, we will use "neuron" and "computational unit" interchangeably. We assume that the preferred orientation space $\Theta = \{\theta_1, \ldots, \theta_N\}$ uniformly partitions the full orientation space $[0, 2\pi)$. I.e., $\theta_j = (j-1)\Delta\theta$, $j = 1, \ldots, N$, where $\Delta\theta = 2\pi/N$. Let h_j, $j = 1, \ldots, N$, denote the total input activity (for example, current) to neuron j in the center ring, with preferred orientation θ_j. The input-output relationship for neurons in the center ring is taken to be threshold linear, such that the activity of neuron j is given by $r_j = [h_j]_+$. The center ring system is given by

$$\tau \dot{h}_j = -h_j + c_{ff} + \frac{1}{N}\sum_{k=1}^{N} W_{jk}^{\text{sym}} r_k + h_j^{CW} + h_j^{CCW}, \quad j = 1, \ldots, N, \tag{S1}$$

where dot notation is used for the time derivative, $\tau > 0$ is the neural time constant, $c_{ff} > 0$ is a constant feedforward input to all units in the center ring, and h_j^*, $* \in \{CW, CCW\}$, indicates the input received from the respective side rings. The weight matrix W^{sym} gives the weights of the recurrent connections in the center ring. We take these weights to be given by

$$W_{jk}^{\text{sym}} = J_I + J_E \cos(\theta_j - \theta_k), \quad j, k = 1, \ldots, N. \tag{S2}$$

The parameters (J_I, J_E) can be set such that this system will generate a population profile that qualitatively looks like a discretely sampled "bump" of activity. We will assume the parameters (J_I, J_E) are within the subset $\Omega = \Omega_{J_I} \times \Omega_{J_E}$ of parameter space for which this is the case (shown as the subset marked "inhomogeneous; stable" in Extended Data Fig 3a). Note that $\Omega \subset (-\infty, 1) \times (2, \infty)$. In the main text we assume $J_I < 0$ for ease of language. This does not affect our results, and the more general case $(J_I, J_E) \in \Omega$ is considered here.

We take the activity in the side ring neurons to be a velocity modulated bump inherited from the center ring that is shifted in orientation space according to whether the ring responds to clockwise (CW) or counter-clockwise (CCW)

movements:

$$\tau_v \dot{h}_j^{CW} = -h_j^{CW} + \frac{v^{CW}}{N}\sum_{k=1}^{N} W_{jk}^{CW} r_k, \qquad W_{jk}^{CW} = \cos(\theta_j - \theta_k - \delta), \quad j,k = 1,\ldots,N, \tag{S3}$$

$$\tau_v \dot{h}_j^{CCW} = -h_j^{CCW} + \frac{v^{CCW}}{N}\sum_{k=1}^{N} W_{jk}^{CCW} r_k, \qquad W_{jk}^{CCW} = \cos(\theta_j - \theta_k + \delta), \quad j,k = 1,\ldots,N, \tag{S4}$$

where τ_v is the neural time constant for the side rings, $v^{CCW} = [v_{\text{in}}]_+$, $v^{CW} = [-v_{\text{in}}]_+$ for angular input velocity v_{in}, and $\delta \in [0,\pi]$ indicates the shift in the inherited bump. Throughout, we will take $\delta = \pi/2$ so that $W^{CW} = -W^{CCW} = W^{\text{asym}}$, where

$$W_{jk}^{\text{asym}} = \sin(\theta_j - \theta_k). \tag{S5}$$

For ease of analytics, we take $\tau_v \to 0$, which reduces the system to a single ring with equations given by

$$\tau \dot{h}_j = -h_j + c_{ff} + \frac{1}{N}\sum_{k=1}^{N}(W_{jk}^{\text{sym}} + v_{\text{in}} W_{jk}^{\text{asym}}) r_k, \quad j = 1,\ldots,N. \tag{S6}$$

Order Equations. Taking the discrete Fourier transform, $H_m = \frac{1}{N}\sum_{j=1}^{N} h_j e^{-im\theta_j}$, of (S6) gives us the order equations:

$$\tau \dot{H}_0 = -H_0 + \frac{J_I}{N}\sum_{k=1}^{N} r_k + c_{ff}, \tag{S7}$$

$$\tau \dot{H}_1 = -H_1 + \frac{1}{2N}\sum_{k=1}^{N}[J_E + v_{\text{in}} i] r_k e^{-i\theta_k}, \tag{S8}$$

$$\tau \dot{H}_m = -H_m, \quad m = 2,\ldots,\lfloor N/2 \rfloor. \tag{S9}$$

Hence, only the DC mode H_0 and the first mode H_1 remain after some initial transients. Note that $\{h_1,\ldots,h_N\}$ is real, and therefore $H_{N-k} = \overline{H}_k$, where $\overline{z}$ denotes the complex conjugate of z. Since $H_1 \in \mathbb{C}$, we let $H_1 = \rho e^{-i\psi}$, and the equations become

$$\tau \dot{H}_0 = -H_0 + \frac{J_I}{N}\sum_{k=1}^{N} r_k + c_{ff}, \tag{S10}$$

$$\tau \dot{\rho} = -\rho + \frac{1}{2N}\sum_{k=1}^{N}[J_E \cos(\theta_k - \psi) + v_{\text{in}} \sin(\theta_k - \psi)] r_k, \tag{S11}$$

$$\tau \rho \dot{\psi} = \frac{1}{2N}\sum_{k=1}^{N}[J_E \sin(\theta_k - \psi) - v_{\text{in}} \cos(\theta_k - \psi))] r_k, \tag{S12}$$

where we assume that we are past any initial transients so that we may ignore (S9). This assumption also allows us to write

$$h_j = H_0 + H_1 e^{i\theta_j} + H_{N-1} e^{-i\theta_j} = H_0 + 2\rho\cos(\theta_j - \psi). \tag{S13}$$

We assume that the parameters $(J_I, J_E) \in \Omega$ so that the population activity appears as a single bump of activity with a proper nonempty subset of neurons active at a single time. Thus, we must have $\rho > 0$. We define the amplitude of the bump relative to the average input activity H_0 to be

$$a = 2\rho, \tag{S14}$$

and we define the width of the bump w by

$$\cos\left(\frac{w}{2}\right) = -\frac{H_0}{a}. \tag{S15}$$

From this definition of bump width, it follows that $h_j > 0$ if and only if $\theta_j \in (\psi - w/2, \psi + w/2)$. With these definitions, we can rewrite (S13) as

$$h_j = a(\cos(\theta_j - \psi) - \cos(w/2)). \tag{S16}$$

We can now uniquely describe the population activity by the relative amplitude a, width w, and orientation ψ of the bump. In what follows, we will refer to this description as the configuration of the population bump, denoted by $\chi = (a, w, \psi)$. Note that we can always recover the individual neuron input activities from the bump configuration through (S16). Let K_{act} denote the set of indices of active neurons in the network. That is,

$$K_{\text{act}} = \{j \mid h_j > 0\} = \{j \mid \theta_j \in (\psi - w/2, \psi + w/2)\}. \tag{S17}$$

We then define the following functions:

$$f_0(w, \psi) = \frac{1}{N} \sum_{k \in K_{\text{act}}} (\cos(\theta_k - \psi) - \cos(w/2)), \tag{S18}$$

$$f_{\text{even}}(w, \psi) = \frac{1}{N} \sum_{k \in K_{\text{act}}} (\cos(\theta_k - \psi) - \cos(w/2)) \cos(\theta_k - \psi), \tag{S19}$$

$$f_{\text{odd}}(w, \psi) = \frac{1}{N} \sum_{k \in K_{\text{act}}} (\cos(\theta_k - \psi) - \cos(w/2)) \sin(\theta_k - \psi). \tag{S20}$$

We can then rewrite (S10) - (S12) as

$$\tau \dot{a} = (-1 + J_E f_{\text{even}}(w, \psi) + v_{\text{in}} f_{\text{odd}}(w, \psi))a, \tag{S21}$$

$$\tau \dot{w} = \frac{2}{\sin(w/2)} \left(J_I f_0(w, \psi) + \frac{c_{ff}}{a} + (J_E f_{\text{even}}(w, \psi) + v_{\text{in}} f_{\text{odd}}(w, \psi)) \cos(w/2) \right), \tag{S22}$$

$$\tau \dot{\psi} = J_E f_{\text{odd}}(w, \psi) - v_{\text{in}} f_{\text{even}}(w, \psi). \tag{S23}$$

Note that in (S22), $\sin(w/2) \neq 0$ since $(J_I, J_E) \in \Omega \Rightarrow w \in (0, 2\pi)$.

Fixed Point Solutions

In this section, we will characterize the stationary fixed point solutions that emerge in the absence of input (i.e., for $v_{\text{in}} = 0$). In this case, (S6) becomes

$$\tau \dot{h}_j = -h_j + c_{ff} + \frac{1}{N} \sum_{k=1}^{N} W_{jk}^{\text{sym}} r_k, \quad j = 1, \ldots, N, \tag{S24}$$

and the order equations, (S21)-(S23), become

$$\tau \dot{a} = (-1 + J_E f_{\text{even}}(w, \psi))a, \tag{S25}$$

$$\tau \dot{w} = \frac{2}{\sin(w/2)} \left(J_I f_0(w, \psi) + \frac{c_{ff}}{a} + J_E f_{\text{even}}(w, \psi) \cos(w/2) \right), \tag{S26}$$

$$\tau \dot{\psi} = J_E f_{\text{odd}}(w, \psi). \tag{S27}$$

Fixed Point Analysis. We perform a fixed point analysis, setting (S25)-(S27) to zero and solving for $\chi^* = (a^*, w^*, \psi^*)$. We will denote fixed point solutions using a superscript $*$ to distinguish them from more general bump configurations. We will replace this superscript with s (stable) or u (unstable) once we determine the stability of the fixed points.

Considering first (S27), this gives us

$$f_{\text{odd}}(w^*, \psi^*) = 0, \tag{S28}$$

since $J_E \in \Omega_{J_E} \subset (2, \infty)$ by assumption. Note that whenever ψ is at a preferred orientation θ_j or is precisely

between two preferred orientations, $f_{\text{odd}}(w, \psi)$ will be the sum of an anti-symmetric function about ψ, and thus

$$\psi^*_{cd} = \frac{1}{2}(\theta_c + \theta_d), \quad c = 1, \ldots, N, \quad d = c, c+1, \tag{S29}$$

will always satisfy (S28), regardless of the value of w^*, where here we take the extension of indices given by $c \equiv c \pmod N$. We then expect the system to have at least $2N$ fixed points: N for orientations ψ^*_{cc} at each of the N preferred orientations, and N for orientations $\psi^*_{c(c+1)}$ precisely between consecutive preferred orientations. This assumes, of course, that there exist corresponding values of a^*_{cd}, w^*_{cd} for which (S25) and (S26) will also equal 0 (which does turn out to be the case). Note that while these $2N$ values of ψ^*_{cd} characterize a set of solutions to (S28), they do not necessarily characterize *all* of the solutions (see Extended Data Fig 3b).

Setting (S25) to zero gives us

$$J_E f_{\text{even}}(w^*, \psi^*) = 1, \tag{S30}$$

since $a^* > 0$ by assumption. Implicit solutions (w^*, ψ^*) to (S30) exist for any ψ^* (given our assumption that $J_E \in \Omega_{J_E}$), indicating a necessary relationship between w^* and ψ^* that is implicitly a function of $1/J_E$ for fixed point solutions (see Extended Data Fig 3c). Specifically, for each ψ^*_{cd} given by (S29), there exists a w^*_{cd} such that (S30) is satisfied. We then need only determine a^* to fully characterize the fixed point solutions to the system. We obtain a^* by setting (S26) to zero and using (S30):

$$a^* = \frac{-c_{ff}}{\cos(w^*/2) + J_I f_0(w^*, \psi^*)}. \tag{S31}$$

Equations (S28),(S30)-(S31) allow us to fully (implicitly) characterize the configuration $\chi^* = (a^*, w^*, \psi^*)$ of the fixed point solutions. Note that, since $a^* > 0$, we must have

$$J_I < \frac{-\cos(w^*/2)}{f_0(w^*, \psi^*)}. \tag{S32}$$

Recall that $f_0(w, \psi)$ is a nonempty sum of positive terms, and thus $f_0(w^*, \psi^*) > 0$.

Local Stability by Linearization The stability of the fixed points identified above can be determined through the linearization of (S25)-(S27). The Jacobian matrix evaluated at the configuration $\chi^*_{cd} = (a^*_{cd}, w^*_{cd}, \psi^*_{cd})$ that satisfies (S29)-(S31) is given by

$$J|_{\chi^*_{cd}} = \frac{1}{\tau}\begin{bmatrix} 0 & \frac{\partial F_a}{\partial w} & 0 \\ \frac{\partial F_w}{\partial a} & \frac{\partial F_w}{\partial w} & 0 \\ 0 & 0 & \frac{\partial F_\psi}{\partial \psi} \end{bmatrix}\Bigg|_{\chi^*_{cd}}, \tag{S33}$$

where $F_a(a, w, \psi) = \tau\dot{a}$, $F_w(a, w, \psi) = \tau\dot{w}$, and $F_\psi(a, w, \psi) = \tau\dot{\psi}$. Thus, one of the eigenvalues of (S33) is given by λ_ψ/τ, where

$$\lambda_\psi = \frac{\partial F_\psi}{\partial \psi} = -1 + \frac{J_E}{N}\sum_{k \in K_{\text{act}}} \sin^2(\theta_k - \psi^*_{cd}). \tag{S34}$$

λ_ψ can be either negative or positive, depending on the orientation ψ^*_{cd} and the value of the parameter J_E (see Extended Data Fig 3d, g). The other two eigenvalues are given by $\lambda_\pm/\tau$, where

$$\lambda_\pm = \frac{1}{2}\left((J_I + J_E)\frac{N_{\text{act}}}{N} - 3 - \lambda_\psi \pm \sqrt{\left((J_I - J_E)\frac{N_{\text{act}}}{N} + \lambda_\psi + 1\right)^2 + 4J_I J_E\left(\frac{1}{N}\sum_{k \in K_{\text{act}}}\cos(\theta_k - \psi^*_{cd})\right)^2}\right). \tag{S35}$$

Here, N_{act} is the number of "active" neurons (i.e., the size of the support of the bump, equal to the number of indices in K_{act}). No matter the sign of λ_ψ, we find that $\lambda_\pm < 0$ as long as (S32) holds (see Extended Data Fig 3e-f,h-i). Thus, the stability of the fixed point χ^*_{cd} is determined by the sign of λ_ψ. When $\lambda_\psi < 0$, χ^*_{cd} is a stable fixed point. When $\lambda_\psi > 0$, χ^*_{cd} is a saddle point, at which the unstable manifold is tangent to the ψ dimension. Given the existence of nonhyperbolic fixed points (when $\lambda_\psi = 0$), we next turn to analyzing an energy function of the system.

Energy Landscape. The energy of the stationary system is given by

$$E = \sum_{j=1}^{N} \int_0^{h_j} h\phi'(h)\, dh - \frac{1}{2N} \sum_{j,k=1}^{N} W_{jk}^{\text{sym}} \phi(h_j)\phi(h_k) - c_{ff} \sum_{j=1}^{N} \phi(h_j), \tag{S36}$$

where $r_k = \phi(h_k)$ is the input-output function [27, 28], here taken to be threshold linear. Note that h_j, $j = 1, \ldots, N$, is bounded, since $(J_I, J_E) \in \Omega$, and thus E is bounded. Since W^{sym} is symmetric, we have

$$\begin{aligned} \dot{E} &= \sum_{j=1}^{N} h_j \phi'(h_j)\dot{h_j} - \frac{1}{N} \sum_{j,k=1}^{N} W_{jk}^{\text{sym}} \phi(h_k)\phi'(h_j)\dot{h_j} - c_{ff} \sum_{j=1}^{N} \phi'(h_j)\dot{h_j} \\ &= \sum_{j=1}^{N} \phi'(h_j)\dot{h_j} \left(h_j - \frac{1}{N} \sum_{k=1}^{N} W_{jk}^{\text{sym}} \phi(h_k) - c_{ff} \right) \\ \Rightarrow \dot{E} &= -\tau \sum_{j=1}^{N} \phi'(h_j)(\dot{h_j})^2 \leq 0. \end{aligned} \tag{S37}$$

Hence, E is non-increasing as a function of time and will converge to a state for which $\dot{E} = 0$. These states correspond to the fixed points of the system. Further, since the unstable fixed points are saddle points, and since the unstable manifold at these saddle points is tangent to the ψ dimension, the energy will converge to a minimum corresponding to a stable fixed point so long as the system does not start at an unstable orientation. From (S16), we can consider the energy E to be a function of the configuration of the bump. That is, $E = E(\chi)$. Using this, we compute the Hessian of the energy in the space of possible bump configurations:

$$H(E) = \begin{bmatrix} \frac{\partial^2 E}{\partial a^2} & \frac{\partial^2 E}{\partial a \partial w} & \frac{\partial E}{\partial a \partial \psi} \\ \frac{\partial^2 E}{\partial a \partial w} & \frac{\partial^2 E}{\partial w^2} & \frac{\partial^2 E}{\partial w \partial \psi} \\ \frac{\partial^2 E}{\partial a \partial \psi} & \frac{\partial^2 E}{\partial w \partial \psi} & \frac{\partial^2 E}{\partial \psi^2} \end{bmatrix}. \tag{S38}$$

For any $x, y \in \{a, w, \psi\}$, we have

$$\frac{\partial^2 E}{\partial x \partial y} = \sum_{j \in K_{\text{act}}} \left(\frac{\partial h_j}{\partial x} - \frac{1}{N} \sum_{k \in K_{\text{act}}} W_{jk}^{\text{sym}} \frac{\partial h_k}{\partial x} \right) \frac{\partial h_j}{\partial y} + \sum_{j \in K_{\text{act}}} \left(h_j - c_{ff} - \frac{1}{N} \sum_{k \in K_{\text{act}}} W_{jk}^{\text{sym}} h_k \right) \frac{\partial^2 h_j}{\partial x \partial y}. \tag{S39}$$

From (S16), for $j = 1, \ldots, N$, we have

$$\frac{\partial h_j}{\partial a} = \cos(\theta_j - \psi) - \cos(w/2), \qquad \frac{\partial h_j}{\partial w} = \frac{a}{2}\sin(w/2), \qquad \frac{\partial h_j}{\partial \psi} = a\sin(\theta_j - \psi), \tag{S40}$$

$$\frac{\partial^2 h_j}{\partial a^2} = 0, \qquad \frac{\partial^2 h_j}{\partial w^2} = \frac{a}{4}\cos(w/2), \qquad \frac{\partial^2 h_j}{\partial \psi^2} = -a\cos(\theta_j - \psi), \tag{S41}$$

$$\frac{\partial^2 h_j}{\partial a \partial \psi} = \sin(\theta_j - \psi), \qquad \frac{\partial^2 h_j}{\partial a \partial w} = \frac{1}{2}\sin(w/2), \qquad \frac{\partial^2 h_j}{\partial w \partial \psi} = 0. \tag{S42}$$

Evaluating (S39) for different pairs of $x, y \in \{a, w, \psi\}$, we see that both $\frac{\partial^2 E}{\partial a \partial \psi}$ and $\frac{\partial^2 E}{\partial w \partial \psi}$ are summations over an anti-symmetric function about $\psi = \psi^*_{cd}$, for ψ^*_{cd} given in (S29). As a result, if we evaluate the Hessian of the energy at a bump configuration $\chi = (a, w, \psi^*_{cd})$ with orientation ψ^*_{cd}, we get

$$H(E)|_\chi = \left. \begin{bmatrix} \frac{\partial^2 E}{\partial a^2} & \frac{\partial^2 E}{\partial a \partial w} & \frac{\partial E}{\partial a \partial \psi} \\ \frac{\partial^2 E}{\partial a \partial w} & \frac{\partial^2 E}{\partial w^2} & \frac{\partial^2 E}{\partial w \partial \psi} \\ \frac{\partial^2 E}{\partial a \partial \psi} & \frac{\partial^2 E}{\partial w \partial \psi} & \frac{\partial^2 E}{\partial \psi^2} \end{bmatrix} \right|_\chi = \left. \begin{bmatrix} \frac{\partial^2 E}{\partial a^2} & \frac{\partial^2 E}{\partial a \partial w} & 0 \\ \frac{\partial^2 E}{\partial a \partial w} & \frac{\partial^2 E}{\partial w^2} & 0 \\ 0 & 0 & \frac{\partial^2 E}{\partial \psi^2} \end{bmatrix} \right|_\chi. \tag{S43}$$

Thus, at ψ^*_{cd}, the Hessian has the eigenpair $\left(\frac{\partial^2 E}{\partial \psi^2}\Big|_{\chi}, [0,0,1]^T \right)$, indicating that near ψ^*_{cd}, the curvature of the energy as a function of orientation is given by $\frac{\partial^2 E}{\partial \psi^2}\Big|_{\chi}$. Consider then the fixed point of the system at this orientation, $\chi^*_{cd} = (a^*_{cd}, w^*_{cd}, \psi^*_{cd})$. The eigenvalue of the Hessian of the energy at this fixed point is

$$\begin{aligned}
\frac{\partial^2 E}{\partial \psi^2}\Big|_{\chi^*_{cd}} &= a^{*2}_{cd} \sum_{j \in K_{\text{act}}} \left(\sin(\theta_j - \psi^*_{cd}) - \frac{1}{N} \sum_{k \in K_{\text{act}}} W^{\text{sym}}_{jk} \sin(\theta_k - \psi^*_{cd}) \right) \sin(\theta_j - \psi^*_{cd}) \\
&= a^{*2}_{cd} \sum_{j \in K_{\text{act}}} \left(\sin(\theta_j - \psi^*_{cd}) - \frac{1}{N} \sum_{k \in K_{\text{act}}} (J_I + J_E \cos(\theta_j - \theta_k)) \sin(\theta_k - \psi^*_{cd}) \right) \sin(\theta_j - \psi^*_{cd}) \\
&= a^{*2}_{cd} \sum_{j \in K_{\text{act}}} \Bigg(\sin(\theta_j - \psi^*_{cd}) - \frac{J_E}{N} \sum_{k \in K_{\text{act}}} \big(\cos(\theta_j - \psi^*_{cd}) \cos(\theta_k - \psi^*_{cd}) \\
&\qquad + \sin(\theta_j - \psi^*_{cd}) \sin(\theta_k - \psi^*_{cd}) \big) \sin(\theta_k - \psi^*_{cd}) \Bigg) \sin(\theta_j - \psi^*_{cd}) \\
&= a^{*2}_{cd} \sum_{j \in K_{\text{act}}} \sin^2(\theta_j - \psi^*_{cd}) \left(1 - \frac{J_E}{N} \sum_{k \in K_{\text{act}}} \sin^2(\theta_k - \psi^*_{cd}) \right). \qquad \text{(S44)}
\end{aligned}$$

This indicates that the curvature of the energy around fixed points χ^*_{cd} is a function of the strength of local excitation in the system, J_E. The stability of these fixed points can be determined directly from the sign of (S44), and it can further be tuned between being stable and unstable by proper modulation of J_E. Most importantly, however, by setting

$$\frac{1}{J^*_E} = \frac{1}{N} \sum_{k \in K_{\text{act}}} \sin^2(\theta_k - \psi^*_{cd}), \qquad \text{(S45)}$$

we can actually force the curvature as a function of orientation about χ^*_{cd} to zero, thereby generating a marginally stable fixed point about χ^*_{cd}. We will denote these marginally stable fixed points by $\chi^{\text{ms}}(\psi) = (a^{\text{ms}}(\psi), w^{\text{ms}}(\psi), \psi)$, where $\psi \in (\psi^*_{cd} - \Delta\psi_{\text{ms}}/2, \psi^*_{cd} + \Delta\psi_{\text{ms}}/2)$, and where $\Delta\psi_{\text{ms}}$ is the width of the interval about ψ^*_{cd} for which the marginally stable solution exists. We will refer to these values J_E that generate marginally stable solutions as "optimal", and we will denote them by J^*_E.

This finding relies on our choice of connectivity W^{sym} (given by (S2)) and nonlinearity $\phi(\,\cdot\,) = [\,\cdot\,]_+$. However, we do expect this finding to generalize to other choices of connectivity and nonlinearity. Assuming W^{sym} is symmetric, the orientations given by (S29) should still correspond to fixed points of the system. The stable subset of these fixed points will again vary depending on the shape of the bump, and hence on the connectivity parameters. If the energy about these fixed points varies smoothly as a function of the connectivity parameters, then there should exist some parameterization that locally flattens the energy with respect to bump orientation to allow for the stability of the fixed points to change. See Extended Data Fig 5 for simulations in networks with different connectivity structures and nonlinearities that suggest these results hold more broadly.

Performance of Optimal Solutions

Here we analyze the marginally stable fixed point solutions that arise when $J_E = J^*_E$. Note that in (S45), J^*_E is truly a function of N and the number of "active" neurons N_{act}, since ψ^*_{cd} in (S29) can be entirely determined via N. Evaluating (S45) for different possible values of N_{act} shows that $J^*_E \in \Omega_{J_E}$ for $N_{\text{act}} = 2, \ldots, N-2$, indicating that there are $N-3$ values of J^*_E that generate marginally stable fixed point solutions. Considering J^*_E as a function of N_{act} and N alone, we can further simplify (S45) to

$$\frac{1}{J^*_{E,N_{\text{act}}}} = \frac{1}{4} + \frac{1}{2N}\left(\tilde{n} + \frac{\sin(\tilde{n}\Delta\theta)}{\sin(\Delta\theta)} \right), \quad \tilde{n} = N_{\text{act}} - \frac{N}{2}, \qquad \text{(S46)}$$

highlighting that the inverse of these optimal J^*_E symmetrically fill in the inverse parameter space ($\Omega^{-1}_{J_E} = \{1/J_E | J_E \in \Omega_{J_E}\}$) about $J^*_E = 4$ (corresponding to $N_{\text{act}} = N/2$ for N even) as N increases (Fig 2d).

Dynamics in the Absence of Input Velocity. To characterize the performance of the marginally stable solutions in the absence of velocity input (i.e., $v_{\rm in} = 0$), we continue to use the energy landscape of the system. Specifically, we numerically calculate the eigenvalues and eigenvectors of the Hessian as a function of bump orientation. For each $\psi \in [0, 2\pi)$, we compute w via (S30) and a via (S31), and then we numerically calculate the eigenvalues and eigenvectors of $H(E)$ at $\chi = (a, w, \psi)$. Proceeding in this way, we find that the Hessian has a zero eigenvalue across all orientations, with corresponding eigenvectors pointing in directions that include a change in orientation (Extended Data Fig 4). The existence of these zero eigenvalues, together with the fact that they appear when the Hessian is evaluated at χ that satisfies (S30)-(S31), indicates that the optimal values of local excitation J_E^* not only locally flatten the energy landscape about χ_{cd}^*, but also generate a flat basin across all orientations $\psi \in [0, 2\pi)$, with $a^{\rm ms}(\psi)$ and $w^{\rm ms}(\psi)$ varying appropriately to satisfy (S28),(S30)-(S31). Thus, $\Delta\psi_{\rm ms} = \Delta\theta$, and these values J_E^* actually generate ring attractor solutions, allowing systems with as few as $N = 4$ neurons to accurately encode any orientation ψ within the continuous interval $[0, 2\pi)$.

One striking feature of these optimal solutions (that is, the ring attractor solutions generated by optimal J_E^*) is that the number of active neurons $N_{\rm act}$ remains constant at each orientation $\psi \in [0, 2\pi)$. To maintain this, whenever an active neuron (or neuron in the support of the bump) becomes inactive (or leaves the support), a new neuron becomes active (or joins the support), thereby keeping $N_{\rm act}$ fixed as the orientation changes. This allows us to consider the subsystem of active neurons

$$\tau \dot{h}_j = -h_j + c_{ff} + \frac{1}{N} \sum_{k \in K_{\rm act}} W_{jk}^{\rm sym} h_k, \quad j \in K_{\rm act}, \tag{S47}$$

as a linear system. Taking $W_{N_{\rm act}}^0$ to be the $N_{\rm act} \times N_{\rm act}$ "active submatrix" along the diagonal of $W^0 = \frac{1}{\tau}(-I + W^{\rm sym}/N)$, we can rewrite (S47) as

$$\dot{\vec{h}}_{\rm act} = W_{N_{\rm act}}^0 \vec{h}_{\rm act} + c_{ff}\vec{e}, \tag{S48}$$

where $\vec{h}_{\rm act} \in \mathbb{R}_+^{N_{\rm act}}$ denotes the vector of inputs to the active neurons, $\vec{e} \in \mathbb{R}^{N_{\rm act}}$ is the vector of all ones, $\vec{e} = [1, \dots, 1]^T$, and we let the constant c_{ff} absorb the inverse neural time constant $1/\tau$. We find the leading eigenvalue of $W_{N_{\rm act}}^0$ to be 0 whenever $J_E = J_{E,N_{\rm act}}^*$, $N_{\rm act} = [2, \dots, N-2]$ (Extended Data Fig 6). This indicates that the "active" subsystem generates a line attractor. As a result, one can think of these optimal ring attractor solutions as being line attractors stitched together at each point where the support of the bump changes. Note that the presence of a zero leading eigenvalue in $W_{N_{\rm act}}^0$ when $J_E = J_E^*$ indicates that these "optimal" values of J_E render the system degenerate. Although such degeneracies are unlikely to occur in biology (due to the strict tuning required), we find the degenerate system to be a useful tool in analyzing the "non-optimal" system, for which $J_E \neq J_E^*$.

Dynamics in the Presence of Input Velocity. When $v_{\rm in} \neq 0$, the optimal solutions again keep the number of active neurons approximately constant. As a result, we can once again work in the linear subsystem of active neurons:

$$\dot{\vec{h}}_{\rm act} = W_{N_{\rm act}}^{v_{\rm in}} \vec{h}_{\rm act} + c_{ff}\vec{e}, \tag{S49}$$

where now $W_{N_{\rm act}}^{v_{\rm in}}$ is the $N_{\rm act} \times N_{\rm act}$ "active submatrix" along the diagonal of $W^{v_{\rm in}} = \frac{1}{\tau}(-I + (W^{\rm sym} + v_{\rm in} W^{\rm asym})/N)$, and everything else is as above (note that (S48) is a special case of (S49), with $v_{\rm in} = 0$). Simulating the system for a range of constant $v_{\rm in}$ (where, without loss of generality, we choose $v_{\rm in} > 0$) suggests that the system will linearly integrate the input velocity $v_{\rm in}$ (including extremely small $v_{\rm in}$) as desired (see Fig 2f). This is not surprising given the stationary properties already determined in *Supplemental Information* |*Performance of Optimal Solutions* |*Dynamics in the Absence of Input Velocity*.

Performance of Non-Optimal Solutions

To dissect the performance of non-optimal solutions (that is, the solutions to systems with $J_E \neq J_E^*$), we will continue to use the linearity of the subsystem of active neurons. Unlike the optimal case, however, the number of active neurons $N_{\rm act}$ will vary not only as a function of the strength of local excitation J_E, but also as a function of orientation ψ for any given $J_E \neq J_E^*$. As a result, the dynamics are governed by two different linear subsystems: one with

$N_{\text{act}} = n$ active neurons, and one with $N_{\text{act}} = n + 1$ active neurons. As found below, these will correspond to a stable and unstable system, respectively. The value of $n \in \{1, \ldots, N-2\}$ will depend on J_E and can be determined by

$$n(J_E) = \begin{cases} N-2, & 2 < J_E < J^*_{E,N-2} \\ \tilde{n}, & J^*_{E,\tilde{n}+1} < J_E < J^*_{E,\tilde{n}},\ \tilde{n} \in \{2, \ldots, N-3\} \\ 1, & J^*_{E,2} < J_E < \infty \end{cases} . \tag{S50}$$

We note that values of J_E at either extreme (i.e., $J_E > J^*_{E,2}$ and $2 < J_E < J^*_{E,N-2}$) result in solutions that might be biologically implausible. At one end of this extreme, $J_E > J^*_{E,2}$, the system would have to encode a subset of orientations with only a single active neuron. At the other end, $2 < J_E < J^*_{E,N-2}$, the system would be required to keep J_E tightly tuned in order to prevent the evolution to a homogeneous solution (when $J_E < 2$), since the interval $(2, J^*_{E,N-2})$ is small and becomes increasingly so as network size N increases. For this reason, we focus our analysis in the main text to intermediate values of $J_E \in [J^*_{E,N-2}, J^*_{E,2}]$. Mathematically, these analyses can be extended, and we treat the more general case ($J_E \in (2, \infty) = \Omega_{J_E}$) in this supplement.

Due to the rotational invariance of $W^{v_{\text{in}}}$, $W^{v_{\text{in}}}_{N_{\text{act}}}$ will not change for fixed N_{act}, regardless of which subset of contiguous subset of neurons is active. Hence, in what follows, we will analyze the system (S49) over a single angular unit of length $\Delta\theta$. The transition from $N_{\text{act}} = n$ to $N_{\text{act}} = n+1$ active neurons (or vice versa) will change the eigenvalues of $W^{v_{\text{in}}}_{N_{\text{act}}}$, thereby changing the underlying system dynamics and trajectory that the bump orientation ψ traces out over time. Since the dynamics of the active neurons are governed by the linear system (S49), we expect the bump orientation in these two different regimes to be exponential in nature. We therefore take

$$\psi_s(t') = \alpha_s + \beta_s \exp(\lambda_s t'), \tag{S51}$$

$$\psi_u(t) = \alpha_u + \beta_u \exp(\lambda_u t) \tag{S52}$$

to represent the bump orientation in the stable and unstable regimes, respectively. In what follows, we will first analyze the drift of the bump orientation when $v_{\text{in}} = 0$ to determine the drift rates λ_s, λ_u and to identify the orientation $\psi_{\Delta n}$ at which the bump transitions between regimes. We then investigate the effect of small $v_{\text{in}} \neq 0$ for which the values of $\lambda_s, \lambda_u, \psi_{\Delta n}$ are approximately unchanged (see *Methods* | *Model Analytics* | *Small Velocity Approximation*).

Dynamics in the Absence of Input Velocity. When $v_{\text{in}} = 0$, the bump ψ will drift away from unstable orientations and toward a stable one. In what follows, we consider the stable and unstable orientations, ψ^s and ψ^u, within a single angular unit. Without loss of generality, we will assume $\psi^s < \psi^u$. Note that, for any non-optimal J_E, the stable and unstable orientations are given by (S29), with $c = d$ for one orientation, and $c = d+1$ for the other. Hence, we can take $\psi^u = \psi^s + \Delta\theta/2$.

When ψ is sufficiently close to ψ^s, the system will be in the stable regime, and ψ will be given by (S51). Further, we should have $\psi_s \to \psi^s$ as $t \to \infty$. Assuming $\lambda_s < 0$, we then have $\alpha_s = \psi^s$. Suppose in this regime there are n_s active neurons. Then, from (S27) we have

$$\tau \beta_s \lambda_s e^{t\lambda_s} = J_E f_{\text{odd}}(w, \psi^s + \beta_s e^{t\lambda_s}). \tag{S53}$$

Consider

$$\begin{aligned} f_{\text{odd}}(w, \psi^s + \beta_s e^{t\lambda_s}) &= \frac{1}{N} \sum_{k \in K_{\text{act}}} \Big(\cos\left(\theta_k - (\psi^s + \beta_s e^{\lambda_s t})\right) - \cos(w/2) \Big) \sin\left(\theta_k - (\psi^s + \beta_s e^{\lambda_s t})\right) \\ &= \frac{1}{N} \sum_{k \in K_{\text{act}}} \left(\cos(\theta_k - \psi^s) \cos(\beta_s e^{\lambda_s t}) + \sin(\theta_k - \psi^s) \sin(\beta_s e^{\lambda_s t}) - \cos(w/2) \right) \\ &\qquad \left(\sin(\theta_k - \psi^s) \cos(\beta_s e^{\lambda_s t}) - \cos(\theta_k - \psi^s) \sin(\beta_s e^{\lambda_s t}) \right). \end{aligned}$$

Since ψ^s is given by (S29), the summations over odd functions centered at ψ^s will cancel themselves out (being

anti-symmetric about ψ^s). This gives us

$$f_{\text{odd}}(w, \psi^s + \beta_s e^{t\lambda_s}) = -\frac{\sin(\beta_s e^{\lambda_s t})}{N} \sum_{k \in K_{\text{act}}} \big[\big(\cos(\beta_s e^{\lambda_s t}) \cos(\theta_k - \psi^s) - \cos(w/2) \big) \cos(\theta_k - \psi^s) - \cos(\beta_s e^{\lambda_s t}) \sin^2(\theta_k - \psi^s) \big].$$

Taking t large, we use small angle approximations to get

$$f_{\text{odd}}(w, \psi^s + \beta_s e^{t\lambda_s}) \approx \beta_s e^{\lambda_s t} \left(\frac{1}{J^*_{E,n_s}} - f_{\text{even}}(w, \psi^s) \right).$$

Assuming $w \approx w^s$, where $\chi^s = (a^s, w^s, \psi^s)$ is the stable fixed point associated with ψ^s, and using (S30) and (S53), we find

$$\lambda_s \approx \frac{1}{\tau} \left(\frac{J_E}{J^*_{E,n_s}} - 1 \right). \tag{S54}$$

Thus, the drift rate λ_s will depend on how close J_E is to the optimal value J^*_{E,n_s}, with closer J_E corresponding to slower drift. Note that our assumption that $\lambda_s < 0$ requires

$$J_E < J^*_{E,n_s}. \tag{S55}$$

Similarly, when ψ is sufficiently close to ψ^u, the system will be in the unstable regime, and ψ will be given by (S52). If we now take $t \to -\infty$, we should have $\psi_u \to \psi^u$. Assuming now $\lambda_u > 0$, this indicates that $\alpha_u = \psi^u$. Using the same types of approximations as above, we find

$$\lambda_u \approx \frac{1}{\tau} \left(\frac{J_E}{J^*_{E,n_u}} - 1 \right). \tag{S56}$$

where n_u is the number of active neurons. The assumptions that $\lambda_u > 0$ requires

$$J_E > J^*_{E,n_u}. \tag{S57}$$

Since $J^*_{E,N-2} < J^*_{E,N-3} < \cdots < J^*_{E,2}$, inequalities (S55) and (S57) imply that $n_u = n_s + 1$. Hence, $n(J_E) = n_s$, as defined above in (S50). Note that, since λ_u was found by evolving the linear dynamics in negative time, this corresponds to the "repulsion" rate. That is, λ_u indicates the rate at which ψ will be pushed away from the unstable orientation ψ^u. Again, we find from (S56) that this rate will depend on how close J_E is to $J^*_{E,n+1}$, being smaller the closer J_E is to this optimal value.

Given the strictly decreasing nature of the optimal $J^*_{E,n}$ as a function of n, and given the relations (S55), (S57) found between J_E and J^*_E when ψ is moving towards a stable orientation ψ^s or away from an unstable orientation ψ^u, respectively, the linear active subsystem will be stable when $N_{\text{act}} = n$ and unstable when $N_{\text{act}} = n + 1$. Numerically computing the leading eigenvalue of W^0_n and W^0_{n+1} confirms this. Further, we find our approximations of these leading eigenvalues, λ_s and λ_u, to be close fits to the numerically computed values (Extended Data Fig 6).

To understand how much these stable and unstable dynamics affect the overall trajectories of the bump, we next calculate the orientation $\psi_{\Delta n}$ at which the system will transition between these stable and unstable regimes. To find this transition point, we again consider the stable and unstable orientations, ψ^s and $\psi^u = \psi^s + \Delta\theta/2$, respectively, within a single angular unit with length $\Delta\theta$. We assume the system starts in the unstable regime at some orientation $\psi_u(0) = \psi_0 < \psi^u$, implying $\beta_u = \psi_0 - \psi^u$. We define $t = t_{\Delta n}$ to be the time when the orientation reaches this transition point from the unstable regime, and we define $t' = 0$ to be the time when the system crosses into the stable regime. This gives

$$\psi_u(t_{\Delta n}) = \psi_{\Delta n}, \tag{S58}$$

$$\psi_s(0) = \psi_{\Delta n}, \tag{S59}$$

where ψ_u, ψ_s are given by (S52), (S51), respectively, with $\alpha_u = \psi^u = \psi^s + \Delta\theta/2$ and $\alpha_s = \psi^s$. Further, we assume this transition is smooth, requiring

$$\dot{\psi}_u(t_{\Delta n}) = \dot{\psi}_s(0). \tag{S60}$$

Condition (S59) implies that $\beta_s = \psi_{\Delta n} - \psi^s$. We can now rewrite (S51)-(S52) as

$$\psi_s(t') = \psi^s + (\psi_{\Delta n} - \psi^s)\exp(-|\lambda_s|t'), \tag{S61}$$

$$\psi_u(t) = \psi^u + (\psi_0 - \psi^u)\exp(\lambda_u t). \tag{S62}$$

Conditions (S58) and (S60) imply that:

$$\begin{aligned}
\psi_u(t_{\Delta n}) = \psi_{\Delta n} &\implies \psi_{\Delta n} = \psi^s + \frac{\Delta\theta}{2} + \beta_u \exp(\lambda_u t_{\Delta n}) \\
&\implies \beta_u \exp(\lambda_u t_{\Delta n}) = \psi_{\Delta n} - \left(\psi^s + \frac{\Delta\theta}{2}\right), \\
\dot{\psi}_u(t_{\Delta n}) = \dot{\psi}_s(0) &\implies \beta_u \lambda_u \exp(\lambda_u t_{\Delta n}) = -(\psi_{\Delta n} - \psi^s)|\lambda_s| \\
&\implies \lambda_u \left(\psi_{\Delta n} - \left(\psi^s + \frac{\Delta\theta}{2}\right)\right) = -(\psi_{\Delta n} - \psi^s)|\lambda_s| \\
&\implies \psi_{\Delta n} = \psi^s + \frac{\Delta\theta}{2}\left(\frac{1}{1 + |\lambda_s|/\lambda_u}\right).
\end{aligned} \tag{S63}$$

The stable regime spans a width $\psi_{\Delta n} - \psi^s$ on either side of the stable fixed point ψ^s, and thus the total width $\Delta\theta_s$ of the stable regime is:

$$\Delta\theta_s = 2(\psi_{\Delta n} - \psi^s) = \Delta\theta\left(\frac{1}{1 + |\lambda_s|/\lambda_u}\right). \tag{S64}$$

The width $\Delta\theta_u$ of the unstable regime is then:

$$\Delta\theta_u = \Delta\theta - \Delta\theta_s = \Delta\theta\left(\frac{1}{1 + \lambda_u/|\lambda_s|}\right). \tag{S65}$$

The bump will cross from the unstable regime into the stable regime at a time $t_{\Delta n}$ that satisfies $\psi_u(t_{\Delta n}) = \psi_{\Delta n}$. Solving (S62) for $t_{\Delta n}$ yields

$$t_{\Delta n} = \frac{1}{\lambda_u}\log\left(\frac{\Delta\theta_u}{2(\psi^u - \psi_0)}\right). \tag{S66}$$

Equations (S61)-(S62) can similarly be used to determine the total drift time τ_d that it takes for the bump to drift from within ε_u of an unstable fixed point to within ε_s of a stable fixed point. This is given by the time it takes for the bump to cross into the stable regime (given by (S66) with $\psi_0 = \psi^u(0) - \varepsilon_u$), plus the additional time t_s that it takes for the bump to cross the stable regime, where t_s satisfies $\psi_s(t_s) = \psi^s(0) + \varepsilon_s$. Solving (S61) for t_s yields a total drift time of:

$$\tau_d = \frac{1}{|\lambda_u|}\log\left(\frac{\Delta\theta_u}{2\varepsilon_u}\right) + \frac{1}{|\lambda_s|}\log\left(\frac{\Delta\theta_s}{2\varepsilon_s}\right). \tag{S67}$$

Taking $\varepsilon_u = \Delta\theta_u/2e$ and $\varepsilon_s = \Delta\theta_s/2e$, (S67) reduces to:

$$\tau_d = \frac{1}{|\lambda_u|} + \frac{1}{|\lambda_s|}. \tag{S68}$$

Note that this corresponds to the time it takes the bump to travel an angular distance $\Delta\psi_d = (1 - 1/e)\Delta\theta/2$. In the

main text, we used $\Delta\psi_d/\tau_d$ as a measure of the net drift speed, which can be expressed as

$$|\lambda_d| = \frac{\Delta\psi_d}{\tau_d} = \left(1 - \frac{1}{e}\right)\frac{\Delta\theta}{2}\frac{|\lambda_u||\lambda_s|}{|\lambda_u| + |\lambda_s|} \tag{S69}$$

$$= c\Delta\theta_s|\lambda_s| \tag{S70}$$

$$= c\Delta\theta_u|\lambda_u|, \tag{S71}$$

where $c = (e-1)/2e$ is a constant.

Dynamics in the Presence of Small Input Velocity In the presence of small velocity input v_{in}, the stable and unstable drift rates remain approximately unchanged (see *Methods* | *Model Analytics* | *Small Velocity Approximation*), and thus so too does the location $\psi_{\Delta n}$ of the boundary between stable and unstable regimes (see (S63)). However, the locations of the stable and unstable orientations will change with v_{in}. That is, $\psi^s = \psi^s(v_{\text{in}})$ and $\psi^u = \psi^u(v_{\text{in}})$. The stable and unstable fixed point orientations referenced above and given by (S29) are then $\psi^s(0)$ and $\psi^u(0)$, respectively. To determine the new orientations of these fixed points, we consider a bump that begins at the stable orientation $\psi^s(0)$ with an initial bump velocity v_{in}. Without loss of generality, we assume that $v_{\text{in}} > 0$ and thus drives the bump toward $\psi^u(0) = \psi^s(0) + \Delta\theta/2$. The bump orientation will again be governed by (S51)-(S52), but with a modified set of initial conditions:

$$\dot{\psi}_s(0) = v_{\text{in}}, \tag{S72}$$

$$\psi_s(0) = \psi^s(0), \tag{S73}$$

$$\dot{\psi}_s(t_{\Delta n}) = \dot{\psi}_u(0), \tag{S74}$$

$$\psi_s(t_{\Delta n}) = \psi_u(0) = \psi_{\Delta n}, \tag{S75}$$

where $t' = t_{\Delta n}$ and $t = 0$ are now the times that $\psi_s = \psi_{\Delta n}$ and $\psi_u = \psi_{\Delta n}$, respectively. Initial condition (S72) implies that $\beta_s = -v_{\text{in}}/|\lambda_s|$, while initial condition (S73) implies that $\alpha_s = \psi^s(0) + v_{\text{in}}/|\lambda_s|$. Initial conditions (S74)-(S75) further imply that:

$$\begin{aligned}
\dot{\psi}_s(t_{\Delta n}) = \dot{\psi}_u(0) &\implies v_{\text{in}} \exp(-|\lambda_s|t_{\Delta n}) \;= \lambda_u\beta_u, \\
\psi_s(t_{\Delta n}) = \psi_{\Delta n} &\implies \psi^s(0) + \frac{v_{\text{in}}}{|\lambda_s|}\Big(1 - \exp(-|\lambda_s|t_{\Delta n})\Big) = \psi_{\Delta n} \\
&\implies \psi^s(0) + \frac{v_{\text{in}}}{|\lambda_s|}\left(1 - \frac{\lambda_u\beta_u}{v_{\text{in}}}\right) = \psi_{\Delta n} \\
&\implies \beta_u = \frac{v_{\text{in}}}{\lambda_u} - (\psi_{\Delta n} - \psi^s(0))\frac{|\lambda_s|}{\lambda_u} \\
&\implies \beta_u = \frac{v_{\text{in}}}{\lambda_u} - \left(\frac{\Delta\theta}{2} - (\psi_{\Delta n} - \psi^s(0))\right) \\
&\implies \beta_u = \frac{v_{\text{in}}}{\lambda_u} - (\psi^u(0) - \psi_{\Delta n}), && \text{(S76)} \\
\psi_u(0) = \psi_{\Delta n} &\implies \alpha_u + \beta_u = \psi_{\Delta n} \\
&\implies \alpha_u = \psi_{\Delta n}\left(1 + \frac{|\lambda_s|}{\lambda_u}\right) - \psi^s(0)\frac{|\lambda_s|}{\lambda_u} - \frac{v_{\text{in}}}{\lambda_u} \\
&\implies \alpha_u = \psi^s(0) + \frac{\Delta\theta}{2} - \frac{v_{\text{in}}}{\lambda_u} \\
&\implies \alpha_u = \psi^u(0) - \frac{v_{\text{in}}}{\lambda_u}. && \text{(S77)}
\end{aligned}$$

Together, this yields:

$$\psi_s(t') = \psi^s(0) + \frac{v_{\rm in}}{|\lambda_s|}\left(1 - \exp(-|\lambda_s|t')\right), \tag{S78}$$

$$\psi_u(t) = \psi^u(0) - \frac{v_{\rm in}}{\lambda_u} + \left(\frac{v_{\rm in}}{\lambda_u} - (\psi^u(0) - \psi_{\Delta n})\right)\exp(\lambda_u t). \tag{S79}$$

The bump will cross from the stable to the unstable regimes at a time $t_{\Delta n}$, where

$$t_{\Delta n} = \frac{1}{|\lambda_s|}\log\left(\frac{1}{1 - |\lambda_s|\Delta\theta_s/(2v_{\rm in})}\right) \tag{S80}$$

can be determined from (S78).

In the limit that $t \to -\infty$, the bump will be driven toward (and hence, in forward time, away from) an unstable fixed point with orientation

$$\psi^u(v_{\rm in}) = \psi^u(0) - v_{\rm in}/\lambda_u. \tag{S81}$$

Similarly, in the limit that $t' \to \infty$, the bump will be driven toward a stable fixed point with orientation

$$\psi^s(v_{\rm in}) = \psi^s(0) + v_{\rm in}/|\lambda_s|. \tag{S82}$$

For $v_{\rm in} = 0$, the stable and unstable fixed point orientations are located at $\psi^s = \psi^s(0)$ and $\psi^u = \psi^u(0)$ given by (S29), as above. As $v_{\rm in}$ increases, both fixed point orientations shift toward the boundary between the stable and unstable regimes at $\psi_{\Delta n}$ (the stable orientation shifts in the same direction as $v_{\rm in}$, while the unstable orientation shifts in the opposite direction as $v_{\rm in}$). The stable fixed point orientation will reach this boundary at a threshold velocity $v_{\rm thresh}$ given by:

$$\begin{aligned} v_{\rm thresh} &= |\lambda_s|(\psi_{\Delta n} - \psi^s(0)) \\ &= |\lambda_d|/2c, \end{aligned} \tag{S83}$$

where $c = (e-1)/2e$, as above, and where we have used (S70) to express the threshold velocity in terms of the net drift speed $|\lambda_d|$. At the threshold velocity, the unstable fixed point orientation will have also shifted to this same boundary:

$$\begin{aligned} \psi^u(v_{\rm thresh}) &= \psi^u(0) - v_{\rm thresh}/\lambda_u \\ &= \psi^s(0) + \frac{\Delta\theta}{2} - \frac{|\lambda_s|}{\lambda_u}(\psi_{\Delta n} - \psi^s(0)) \\ &= \psi^s(0) + \frac{1}{2}\left(\Delta\theta - \Delta\theta_u\right) \\ &= \psi_{\Delta n}. \end{aligned}$$

Above this threshold velocity, the location of the stable fixed point orientation is beyond the boundary of the stable regime. As a result, the bump will be pulled toward this orientation but will never reach it; instead, the bump will cross the boundary into the unstable regime, where it will be pushed away from an unstable fixed point. The orientation of this unstable fixed point is similarly beyond the boundary of the unstable regime, and pushes the bump from behind. As a result, the bump will be pulled toward and pushed away from stable and unstable orientations that it can't reach. This will cause the bump to decelerate and accelerate as it travels through the stable and unstable regimes, respectively. The bump will be moving the slowest as it passes the boundary from the stable into the unstable regime (i.e., at $\psi_s = \psi_{\Delta n}$); beyond this point, it will begin accelerating away from the unstable fixed point. To find the velocity at this point, note that

$$\psi_s(t_{\Delta n}) = \psi_{\Delta n} \implies \psi_{\Delta n} = \psi^s(0) + \frac{v_{\text{in}}}{|\lambda_s|}(1 - \exp(-|\lambda_s| t_{\Delta n}))$$
$$\implies \exp(-|\lambda_s| t_{\Delta n}) = 1 - (\psi_{\Delta n} - \psi^s(0))\frac{|\lambda_s|}{v_{\text{in}}}.$$

The slowest bump velocity $\nu_{\min}$ is thus given by

$$\begin{aligned}
\nu_{\min} = \dot{\psi}_s(t_{\Delta n}) &= v_{\text{in}} \exp(-|\lambda_s| t_{\Delta n}) \\
&= v_{\text{in}} \left(1 - (\psi_{\Delta n} - \psi^s(0))\frac{|\lambda_s|}{v_{\text{in}}}\right) \\
&= v_{\text{in}} - |\lambda_s|(\psi_{\Delta n} - \psi^s(0)) \\
&= v_{\text{in}} - v_{\text{thresh}}.
\end{aligned} \tag{S84}$$

The bump will be moving the fastest as it passes the boundary from the unstable into the stable regime (i.e., at $\psi_u = \psi_{\Delta n} + \Delta\theta_u = 2\psi^u(0) - \psi_{\Delta n}$); beyond this point, it will begin decelerating toward the stable fixed point. To find the velocity at this point, note that

$$\psi_u(t_{\Delta n}) = 2\psi^u(0) - \psi_{\Delta n} + \implies 2\psi^u(0) - \psi_{\Delta n} = \psi^u(0) - \frac{v_{\text{in}}}{\lambda_u} + \left(\frac{v_{\text{in}}}{\lambda_u} - \big(\psi^u(0) - \psi_{\Delta n}\big)\right)\exp(\lambda_u t_{\Delta n})$$
$$\implies \left(\frac{v_{\text{in}}}{\lambda_u} - \big(\psi^u(0) - \psi_{\Delta n}\big)\right)\exp(\lambda_u t_{\Delta n}) = \frac{v_{\text{in}}}{\lambda_u} - \big(\psi_{\Delta n} - \psi^u(0)\big).$$

The fastest bump velocity $\nu_{\max}$ will thus be given by

$$\begin{aligned}
\nu_{\max} = \dot{\psi}_u(t_{\Delta n}) &= \lambda_u \left(\frac{v_{\text{in}}}{\lambda_u} - \big(\psi^u(0) - \psi_{\Delta n}\big)\right)\exp(\lambda_u t_{\Delta n}) \\
&= \lambda_u \left(\frac{v_{\text{in}}}{\lambda_u} - \big(\psi_{\Delta n} - \psi^u(0)\big)\right) \\
&= \lambda_u \left(\frac{v_{\text{in}}}{\lambda_u} + \frac{|\lambda_s|}{\lambda_u}\big(\psi_{\Delta n} - \psi^s(0)\big)\right) \\
&= v_{\text{in}} + v_{\text{thresh}}.
\end{aligned} \tag{S85}$$

In the main text, we define the linearity of integration ℓ to be

$$\ell = \frac{\nu_{\min}}{\nu_{\max}} = \frac{v_{\text{in}} - v_{\text{thresh}}}{v_{\text{in}} + v_{\text{thresh}}}. \tag{S86}$$

Simplified Energy We now summarize the dynamics of the non-optimally tuned system fully characterized above by a function $\tilde{E} = \tilde{E}(\psi)$ that we call the simplified energy of the network. Given the linearity of the subsystem of active neurons, we take this function to be piecewise quadratic with local minimum (maximum) at the orientation corresponding to the fixed point in the stable (unstable) regime. For notation sake, we denote the fixed points given by (S81)-(S82) as $\psi_v^u = \psi^u(v)$, $\psi_v^s = \psi^s(v)$. Define

$$E_s(\psi; v) = \alpha_s(\psi - \psi_v^s)^2 \tag{S87}$$
$$E_u(\psi; v) = \alpha_u(\psi - \psi_v^u)^2 + C \tag{S88}$$

for constants α_s, α_u, and $C = C(v)$. Further, to ensure that ψ_v^s is the minimum of E_s and ψ_v^u is the maximum of E_u, we have $\alpha_s > 0$ and $\alpha_u < 0$. We then define the simplified energy to be

$$\tilde{E}(\psi; v) = \begin{cases} E_s(\psi; v), & \psi \in [\psi_0^s - \Delta\theta_s/2,\ \psi_0^s + \Delta\theta_s/2) \\ E_u(\psi; v), & \psi \in [\psi_0^u - \Delta\theta_u/2,\ \psi_0^u + \Delta\theta_u/2) \end{cases}. \tag{S89}$$

Recall, $\psi_0^u = \psi_0^s + \Delta\theta/2$ and $\Delta\theta_u = \Delta\theta - \Delta\theta_s$ so that

$$\psi_0^u - \Delta\theta_u/2 = \psi_0^s + \Delta\theta/2 - 1/2(\Delta\theta - \Delta\theta_s) = \psi_0^s + \Delta\theta_s/2.$$

To determine the constants α_s, α_u, C, we require $E_{\rm sim}$ to be smooth at the transition between the stable regime, $[\psi_0^s - \Delta\theta_s/2,\ \psi_0^s + \Delta\theta_s/2)$, and the unstable regime, $[\psi_0^u - \Delta\theta_u/2,\ \psi_0^u + \Delta\theta_u/2)$. Hence, for all $v \in \mathbb{R}$, we require

$$E_s(\psi_0^s + \Delta\theta_s/2; v) = E_u(\psi_0^s + \Delta\theta_s/2; v) \tag{S90}$$
$$E_s'(\psi_0^s + \Delta\theta_s/2; v) = E_u'(\psi_0^s + \Delta\theta_s/2; v) \tag{S91}$$

where prime notation denotes the derivative with respect to the first argument. From (S91), we have

$$\begin{aligned} 2\alpha_s(\psi_0^s + \Delta\theta_s/2 - \psi_v^s) &= 2\alpha_u(\psi_0^u - \Delta\theta_u/2 - \psi_v^u) \\ \Rightarrow \alpha_s(\Delta\theta_s/2 - v/|\lambda_s|) &= \alpha_u(-\Delta\theta_u/2 + v/\lambda_u) \\ \Rightarrow \frac{\alpha_s}{\alpha_u} &= -\left(\frac{\lambda_u\Delta\theta_u - 2v}{2\lambda_u}\right)\left(\frac{2|\lambda_s|}{|\lambda_s|\Delta\theta_s - 2v}\right). \end{aligned}$$

From (S64)-(S65), we have $|\lambda_s|\Delta\theta_s = \lambda_u\Delta\theta_u$. Hence,

$$\frac{\alpha_s}{\alpha_u} = -\frac{|\lambda_s|}{\lambda_u}. \tag{S92}$$

Clearly, (S92) is satisfied by

$$\alpha_s = |\lambda_s| = -\lambda_s > 0 \tag{S93}$$
$$\alpha_u = -\lambda_u < 0. \tag{S94}$$

From (S90),

$$\begin{aligned} \alpha_s(\psi_0^s + \Delta\theta_s/2 - \psi_v^s)^2 &= \alpha_u(\psi_0^u - \Delta\theta_u/2 - \psi_v^u)^2 + C \\ \Rightarrow \alpha_s(\Delta\theta_s/2 - v/|\lambda_s|)^2 &= \alpha_u(-\Delta\theta_u/2 + v/\lambda_u)^2 + C \\ \Rightarrow \alpha_s\left(\frac{|\lambda_s|\Delta\theta_s - 2v}{2|\lambda_s|}\right)^2 &= \alpha_u\left(\frac{-\lambda_u\Delta\theta_u + 2v}{2\lambda_u}\right)^2 + C \\ \Rightarrow C &= \frac{1}{4}\left(\frac{\alpha_s}{|\lambda_s|^2} - \frac{\alpha_u}{\lambda_u^2}\right)(\lambda_u\Delta\theta_u - 2v)^2. \end{aligned}$$

From (S92),

$$C = \frac{1}{4}\left(\frac{\alpha_s - \alpha_u}{\lambda_u|\lambda_s|}\right)(\lambda_u\Delta\theta_u - 2v)^2.$$

With (S93)-(S94), we have

$$C = \frac{1}{4}\left(\frac{|\lambda_s| + \lambda_u}{\lambda_u|\lambda_s|}\right)(\lambda_u\Delta\theta_u - 2v)^2 = \frac{1}{4}\left(\frac{\Delta\theta}{\lambda_u\Delta\theta_u}\right)(\lambda_u\Delta\theta_u - 2v)^2.$$

Thus,

$$C = C(v) = \Delta\theta\left(\frac{1}{4}\lambda_u\Delta\theta_u - v + \frac{v^2}{\lambda_u\Delta\theta_u}\right). \tag{S95}$$

Here we've defined $\tilde{E}$ locally for an arbitrary pair of successive stable and unstable regimes. To move around the ring into other stable/unstable regimes, the energy landscape will retain this basic form but must be shifted vertically to ensure continuity (as shown in Fig 3i,j).

To see this, consider the stable regime directly to the right of $[\psi_0^u - \Delta\theta_u/2, \psi_0^u + \Delta\theta_u/2)$. Let $\psi_v^{s,1}$ denote the orientation of the stable fixed point in this regime. The rotational invariance of $W^{v_{\rm in}}$ ensures that $\psi_v^{s,1} = \psi_v^s + \Delta\theta$ (i.e., the orientation of the stable fixed point for the next stable regime is shifted by $\Delta\theta$ from the orientation of

the stable fixed point for the previous stable regime). Hence, this fixed point determines the dynamics for $\psi \in [\psi_0^{s,1} - \Delta\theta_s/2, \psi_0^{s,1} + \Delta\theta_s/2) = [\psi_0^s + \Delta\theta - \Delta\theta_s/2, \psi_0^s + \Delta\theta + \Delta\theta_s/2) = [\psi_0^s - \Delta\theta_s/2, \psi_0^s + \Delta\theta_s/2) + \Delta\theta$. Noting that

$$|\lambda_s|(\psi + \Delta\theta - \psi_v^{s,1})^2 = |\lambda_s|(\psi + \Delta\theta - (\psi_v^s + \Delta\theta))^2 = E_s(\psi)$$

(i.e., the energy is invariant to shifts by $\Delta\theta$) and

$$\psi_0^u + \Delta\theta_u/2 = \psi_0^s + \Delta\theta/2 + 1/2(\Delta\theta - \Delta\theta_s) = \psi_0^s + \Delta\theta - \Delta\theta_s/2 = \psi_0^{s,1} - \Delta\theta_s/2$$

we seek to determine $\hat{C}$ such that

$$E_u(\psi_0^u + \Delta\theta_u/2) = E_s(\psi_0^s - \Delta\theta_s/2) + \hat{C}.$$

Following a similar process as that above to solve for $C(v)$, we find

$$\hat{C} = C(v) - \Delta\theta\left(\frac{1}{4}\lambda_u\Delta\theta_u + v + \frac{v^2}{\lambda_u\Delta\theta_u}\right) = -2v\Delta\theta.$$

Similarly, moving to the pair of stable/unstable regimes directly to the left of $[\psi_0^s - \Delta\theta_s/2, \psi_0^s + \Delta\theta_s/2)$ requires a vertical shift of $+2v\Delta\theta$. Further, each consecutive shift in ψ by $\pm\Delta\theta$ will require an additional vertical shift in the energy by $\mp 2v\Delta\theta$. Hence, in general we have $E(\psi \pm n\Delta\theta) = E(\psi) \mp 2nv\Delta\theta$ for $n \in \mathbb{N}$.

Degradation of Performance as a Function of Local Excitation. In the main text, we consider three different measures of performance: the net drift speed, the threshold input velocity needed to move the bump continuously, and the linearity of integration for input velocities above the threshold value. To characterize how each of these performance measures locally degrades as we tune the local excitation away from an optimal value, we consider the derivative of each performance measure P with respect to J_E when evaluated at an optimal value of local excitation J_E^*:

$$m_P(J_E^*) = \left.\frac{\partial P}{\partial J_E}\right|_{J_E^*}, \tag{S96}$$

where $P \in \{|\lambda_d|, v_{\mathrm{thresh}}, \ell\}$ is one of the net drift speed, threshold velocity, or linearity of integration, respectively. Because each performance measure can be written in terms of the net drift speed, we can rewrite this as

$$m_P(J_E^*) = \left.\frac{\partial P}{\partial|\lambda_d|}\frac{\partial|\lambda_d|}{\partial J_E}\right|_{J_E^*}, \tag{S97}$$

where

$$\frac{\partial v_{\mathrm{thresh}}}{\partial|\lambda_d|} = \frac{1}{2c}, \tag{S98}$$

$$\begin{aligned}\frac{\partial\ell}{\partial|\lambda_d|} &= \frac{-2v_{\mathrm{in}}}{(v_{\mathrm{in}} + v_{\mathrm{thresh}})^2}\frac{\partial v_{\mathrm{thresh}}}{\partial|\lambda_d|} \\ &= \frac{-v_{\mathrm{in}}}{c(v_{\mathrm{in}} + v_{\mathrm{thresh}})^2}.\end{aligned} \tag{S99}$$

In what follows, we first determine $\partial|\lambda_d|/\partial J_E|_{J_E^*}$, and we then use (S97)-(S99) to determine the rate at which performance degrades around the optimal values J_E^*. Taking the derivative of (S69) with respect to J_E, we find

$$\begin{aligned}\frac{\partial|\lambda_d|}{\partial J_E} &= c\Delta\theta\left(\frac{(|\lambda_s| + |\lambda_u|)(|\lambda_s|' + |\lambda_u|') - |\lambda_s||\lambda_u|((|\lambda_s|' + |\lambda_u|')}{(|\lambda_s| + |\lambda_u|)^2}\right) \\ &= c\Delta\theta\left(\frac{|\lambda_u|^2|\lambda_s|' + |\lambda_s|^2|\lambda_u|'}{(|\lambda_s| + |\lambda_u|)^2}\right),\end{aligned} \tag{S100}$$

where $|\lambda|' = \partial|\lambda|/\partial J_E$. From (S54), (S56), we have

$$|\lambda_u| + |\lambda_s| = \frac{J_E}{\tau}\left(\frac{J^*_{E,n} - J^*_{E,n+1}}{J^*_{E,n}J^*_{E,n+1}}\right). \tag{S101}$$

Recall from (S55) and (S57), we have $J^*_{E,n+1} < J_E < J^*_{E,n}$, such that (S101) is positive. Further,

$$|\lambda_s|' = \frac{-1}{\tau J^*_{E,n}}, \tag{S102}$$

$$|\lambda_u|' = \frac{1}{\tau J^*_{E,n+1}}, \tag{S103}$$

such that

$$|\lambda_u|^2|\lambda_s|' + |\lambda_s|^2|\lambda_u|' = \frac{1}{\tau^3}\left(\frac{J^*_{E,n} - J^*_{E,n+1}}{J^*_{E,n}J^*_{E,n+1}}\right)\left(1 - \frac{J_E^2}{J^*_{E,n}J^*_{E,n+1}}\right). \tag{S104}$$

From (S101) and (S104), we find

$$\frac{\partial|\lambda_d|}{\partial J_E} = \frac{c\Delta\theta}{\tau}\left(\frac{J^*_{E,n}J^*_{E,n+1}}{J^*_{E,n} - J^*_{E,n+1}}\right)\left(\frac{1}{J_E^2} - \frac{1}{J^*_{E,n}J^*_{E,n+1}}\right). \tag{S105}$$

Letting J_E approach some optimal value J^*_E from below and above, respectively, we find

$$\lim_{J_E \nearrow J^*_E} \left.\frac{\partial|\lambda_d|}{\partial J_E}\right|_{J_E} = -\frac{c\Delta\theta}{\tau J^*_E}, \tag{S106}$$

$$\lim_{J_E \searrow J^*_E} \left.\frac{\partial|\lambda_d|}{\partial J_E}\right|_{J_E} = \frac{c\Delta\theta}{\tau J^*_E}. \tag{S107}$$

Note that (S106)-(S107) indicate that (S97) will change signs as J_E passes through an optimal value (which is to be expected since the optimal values should correspond to local extrema of our performance measures), but will have a constant magnitude locally about the optimal J^*_E. Thus, for each performance measure P, the magnitude of the slope $|m_P(J^*_E)|$ gives us a local approximation for how performance changes as J_E is tuned away from J^*_E. We can then estimate $P = P(J_E)$ around J^*_E by

$$P(J_E) \approx (J_E - J^*_E)m_P(J^*_E) + P^*, \tag{S108}$$

where $P^* = P(J^*_E)$ indicates the best achievable performance (≈ 0 for $P = |\lambda_d|, v_{\text{thresh}}$, and ≈ 1 for $P = \ell$). Let $P_{\text{desired}} = P^* + \varepsilon_P^{\text{tol}}$ indicate the desired performance threshold. Then, for sufficiently small $|\varepsilon_P^{\text{tol}}|$, the tolerance of the system about J^*_E (defined to be the length of the interval containing J^*_E that satisfies the desired performance threshold) is given approximately by

$$\text{tol}_P(J^*_E) \approx \frac{2\varepsilon_P^{\text{tol}}}{|m_P(J^*_E)|}. \tag{S109}$$

Combining (S106)-(S107) with (S98)-(S99), we have

$$m_{|\lambda_d|}(J^*_E) = \mp\frac{2\pi c}{\tau N J^*_E}, \tag{S110}$$

$$m_{v_{\text{thresh}}}(J^*_E) = \mp\frac{\pi}{\tau N J^*_E}, \tag{S111}$$

$$m_\ell(J^*_E) = \pm\frac{2\pi}{\tau v_{\text{in}} N J^*_E}, \tag{S112}$$

as J_E approaches J^*_E from below and above, respectively. Note that for $P = |\lambda_d|$ or $P = v_{\text{thresh}}$, $m_P(J^*_E)$ decreases away from the optimal value J^*_E, while for $P = \ell$, it increases. Since $|\lambda_d|$ and v_{thresh} are performance measures that should be minimized and ℓ should be maximized (reaching a global maximum at 1), this indicates that, for a desired

performance threshold $P_{\rm desired}$, our estimate will give a lower bound on the actual tolerance about J_E^*:

$$\text{tol}_P(J_E^*) \geq \frac{2\varepsilon_P^{\rm tol}}{|m_P(J_E^*)|}. \tag{S113}$$

Thus, for each performance measure P, the resulting tolerance about J_E^* is proportional to $1/|m_P(J_E^*)|$, which yields

$$\text{net drift speed: } \text{tol}_{|\lambda_d|}(J_E^*) \geq c_{|\lambda_d|} N J_E^*, \tag{S114}$$

$$\text{threshold velocity: } \text{tol}_{v_{\rm thresh}}(J_E^*) \geq c_{v_{\rm thresh}} N J_E^*, \tag{S115}$$

$$\text{linearity of integration: } \text{tol}_{\ell}(J_E^*) \geq c_{\ell} N J_E^* v_{\rm in}, \tag{S116}$$

where

$$c_{|\lambda_d|} = \frac{\tau}{\pi c}\varepsilon_{|\lambda_d|}^{\rm tol}, \tag{S117}$$

$$c_{v_{\rm thresh}} = \frac{2\tau}{\pi}\varepsilon_{v_{\rm thresh}}^{\rm tol}, \tag{S118}$$

$$c_{\ell} = \frac{\tau v_{\rm in}}{\pi}\varepsilon_{\ell}^{\rm tol}. \tag{S119}$$

For a given network size N, we can compute the net volume V of parameter space that satisfies a given performance threshold by summing the tolerances about each optimal values of local excitation:

$$\begin{aligned} V_P &\geq c_P N \sum_{N_{\rm act}=2}^{N-2} J_{E,N_{\rm act}}^* \\ &\geq 2N^2 \sum_{N_{\rm act}=2}^{N-2} \frac{1}{N_{\rm act} - \sin(2\pi N_{\rm act}/N)/\sin(2\pi/N)}, \end{aligned} \tag{S120}$$

where $P \in \{|\lambda_d|, v_{\rm thresh}, \ell\}$ is the given performance measure, and where we have used (S46) to rewrite $J_{E,N_{\rm act}}^*$. The largest contribution to this sum will be for $N_{\rm act} = 2$ (note that this is the only contribution for $N = 4$), which allows us to simplify the lower bound on the net volume to

$$V_P \geq c_P \frac{N^2}{1 - \cos(2\pi/N)}. \tag{S121}$$

References

1. Camperi, M. & Wang, X.-J. A model of visuospatial working memory in prefrontal cortex: recurrent network and cellular bistability. *Journal of Computational Neuroscience* **5,** 383–405 (1998).
2. Compte, A., Brunel, N., Goldman-Rakic, P. S. & Wang, X.-J. Synaptic mechanisms and network dynamics underlying spatial working memory in a cortical network model. *Cerebral Cortex* **10,** 910–923 (2000).
3. Wimmer, K., Nykamp, D. Q., Constantinidis, C. & Compte, A. Bump attractor dynamics in prefrontal cortex explains behavioral precision in spatial working memory. *Nature Neuroscience* **17,** 431–439 (2014).
4. Samsonovich, A. & McNaughton, B. L. Path integration and cognitive mapping in a continuous attractor neural network model. *Journal of Neuroscience* **17,** 5900–5920 (1997).
5. Tsodyks, M. Attractor neural network models of spatial maps in hippocampus. *Hippocampus* **9,** 481–489 (1999).
6. Stringer, S., Trappenberg, T., Rolls, E. & de Araujo, I. Self-organizing continuous attractor networks and path integration: one-dimensional models of head direction cells. *Network* **13,** 217–242 (2002).
7. Boucheny, C., Brunel, N. & Arleo, A. A continuous attractor network model without recurrent excitation: maintenance and integration in the head direction cell system. *Journal of Computational Neuroscience* **18,** 205–227 (2005).
8. Redish, D., Elga, A. & Touretzky, D. A coupled attractor model of the rodent head direction system. *Network: Computation in Neural Systems* **7,** 671–685 (1996).
9. Goodridge, J. & Touretzky, D. Modeling attractor deformation in the rodent head-direction system. *Journal of Neurophysiology* **83,** 3402–3410 (2000).
10. Arleo, A. & Gerstner, W. Spatial orientation in navigating agents: Modeling head-direction cells. *Neurocomputing* **38-40,** 1059–1065 (2001).
11. Sharp, P. E., Blair, H. T. & Cho, J. The anatomical and computational basis of the rat head-direction cell signal. *Trends in Neurosciences* **24,** 289–294 (2001).
12. Xie, X., Hahnloser, R. & Seung, H. Double-ring network model of the head-direction system. *Physical Review E* **66,** 041902 (2002).
13. Song, P. & Wang, X.-J. Angular Path Integration by Moving "Hill of Activity": A Spiking Neuron Model without Recurrent Excitation of the Head-Direction System. *Journal of Neuroscience* **25,** 1002–1014 (2005).
14. Esnaola-Acebes, J. M., Roxin, A. & Wimmer, K. Flexible integration of continuous sensory evidence in perceptual estimation tasks. *Proceedings of the National Academy of Sciences* **119,** e2214441119 (2022).
15. Liu, F. & Wang, X.-J. A common cortical circuit mechanism for perceptual categorical discrimination and veridical judgment. *PLOS Computational Biology* **4,** 1–14 (2008).
16. Furman, M. & Wang, X.-J. Similarity effect and optimal control of multiple-choice decision making. *Neuron* **60,** 1153–1168 (2008).
17. Chaudhuri, R. & Fiete, I. Computational principles of memory. *Nature Neuroscience* **19,** 394–403 (2016).
18. Wang, R. & Kang, L. Multiple bumps can enhance robustness to noise in continuous attractor networks. *PLOS Computational Biology* **18,** e1010547 (2022).
19. Zhang, K. Representation of spatial orientation by the intrinsic dynamics of the head-direction cell ensemble: a theory. *Journal of Neuroscience* **16,** 2112–2126 (1996).
20. Hansel, D & Sompolinsky, H. in *Methods in neuronal modeling: from synapses to networks* (eds Koch, C & Segev, I) 2nd ed. Chap. 13 (Cambridge, MA, 1998).
21. Ben-Yishai, R, Bar-Or, R. L. & Sompolinsky, H. Theory of orientation tuning in visual cortex. *Proceedings of the National Academy of Sciences* **92,** 3844–3848 (1995).
22. Pisokas, I., Heinze, S. & Webb, B. The head direction circuit of two insect species. *eLife* **9,** e53985 (2020).
23. Brody, C. D., Romo, R. & Kepecs, A. Basic mechanisms for graded persistent activity: discrete attractors, continuous attractors, and dynamic representations. *Current Opinion in Neurobiology* **13,** 204–211 (2003).
24. Burak, Y. & Fiete, I. R. Accurate path integration in continuous attractor network models of grid cells. *PLOS Computational Biology* **5,** e1000291 (2009).
25. Kakaria, K. S. & de Bivort, B. L. Ring attractor dynamics emerge from a spiking model of the entire protocerebral bridge. *Frontiers in Behavioral Neuroscience* **11** (2017).
26. Turner-Evans, D. B., Wegener, S., Rouault, H., Franconville, R., Wolff, T., Seelig, J. D., Druckmann, S. & Jayaraman, V. Angular velocity integration in a fly heading circuit. *eLife* **6,** e23496 (2017).
27. Cohen, M. A. & Grossberg, S. Absolute stability of global pattern formation and parallel memory storage by competitive neural networks. *IEEE Transactions on Systems, Man, and Cybernetics* **SMC-13,** 815–826 (1983).
28. Hopfield, J. J. Neurons with graded response have collective computational properties like those of two-state neurons. *Proceedings of the National Academy of Sciences* **81,** 3088–3092 (1984).
