# Supplementary material for: Maintaining and updating accurate internal representations of continuous variables with a handful of neurons
Source: Nat Neurosci. 2024 Oct 3;27(11):18. doi: 10.1038/s41593-024-01766-5 (PMC11537979; doi:10.1038/s41593-024-01766-5)
Supplement: Supplementary file 2 — Reporting Summary [file 41593_2024_1766_MOESM2_ESM.pdf]

nature portfolio

Corresponding author(s): Ann M Hermundstad

Last updated by author(s): July 24, 2024

Reporting Summary

Nature Portfolio wishes to improve the reproducibility of the work that we publish. This form provides structure for consistency and transparency in reporting. For further information on Nature Portfolio policies, see our Editorial Policies and the Editorial Policy Checklist.

Statistics

For all statistical analyses, confirm that the following items are present in the figure legend, table legend, main text, or Methods section.

n/a	Confirmed	
☐	☒	The exact sample size (*n*) for each experimental group/condition, given as a discrete number and unit of measurement
☐	☒	A statement on whether measurements were taken from distinct samples or whether the same sample was measured repeatedly
☐	☒	The statistical test(s) used AND whether they are one- or two-sided<br>*Only common tests should be described solely by name; describe more complex techniques in the Methods section.*
☒	☐	A description of all covariates tested
☒	☐	A description of any assumptions or corrections, such as tests of normality and adjustment for multiple comparisons
☐	☒	A full description of the statistical parameters including central tendency (e.g. means) or other basic estimates (e.g. regression coefficient) AND variation (e.g. standard deviation) or associated estimates of uncertainty (e.g. confidence intervals)
☐	☒	For null hypothesis testing, the test statistic (e.g. *F*, *t*, *r*) with confidence intervals, effect sizes, degrees of freedom and *P* value noted<br>*Give P values as exact values whenever suitable.*
☒	☐	For Bayesian analysis, information on the choice of priors and Markov chain Monte Carlo settings
☒	☐	For hierarchical and complex designs, identification of the appropriate level for tests and full reporting of outcomes
☒	☐	Estimates of effect sizes (e.g. Cohen's *d*, Pearson's *r*), indicating how they were calculated

Our web collection on statistics for biologists contains articles on many of the points above.

Software and code

Policy information about availability of computer code

Data collection	All two-photon calcium imaging experiments were performed using a custom microscope controlled with ScanImage (version 2022; Vidrio Technologies). Behavioral data and imaging timestamps were collected using WaveSurfer (version 0.947).
Data analysis	All code was written in Matlab R2020a. The code is freely available at https://doi.org/10.5281/zenodo.12789923 and is maintained at https://github.com/HermundstadLab/DiscreteRingAttractor.

For manuscripts utilizing custom algorithms or software that are central to the research but not yet described in published literature, software must be made available to editors and reviewers. We strongly encourage code deposition in a community repository (e.g. GitHub). See the Nature Portfolio guidelines for submitting code & software for further information.

Data

Policy information about availability of data

All manuscripts must include a data availability statement. This statement should provide the following information, where applicable:

- Accession codes, unique identifiers, or web links for publicly available datasets
- A description of any restrictions on data availability
- For clinical datasets or third party data, please ensure that the statement adheres to our policy

Data is freely available at https://doi.org/10.25378/janelia.26169355.

Human research participants

Policy information about studies involving human research participants and Sex and Gender in Research.

Reporting on sex and gender	N/A
Population characteristics	N/A
Recruitment	N/A
Ethics oversight	N/A

Note that full information on the approval of the study protocol must also be provided in the manuscript.

Field-specific reporting

Please select the one below that is the best fit for your research. If you are not sure, read the appropriate sections before making your selection.

☒ Life sciences ☐ Behavioural & social sciences ☐ Ecological, evolutionary & environmental sciences

For a reference copy of the document with all sections, see nature.com/documents/nr-reporting-summary-flat.pdf

Life sciences study design

All studies must disclose on these points even when the disclosure is negative.

Sample size	Calcium imaging experiments in behaving flies involve tethering, dissections and positioning on the ball. These steps are each subject to some variability. In addition, fly behavior on the ball is variable. These factors, rather than statistical methods, motivated us to perform experiments in 10 flies with long trial durations (5 trials of 20 minutes each). We recorded 100 minutes of data without visual stimuli for each fly, which we assume to be sufficient for the types of analyses we performed. A sample size of 10 flies was chosen to be consistent with previous studies of this circuit (e.g. Seelig and Jayaraman, 2015).
Data exclusions	We excluded any data collected beyond 100 minutes for consistency and to exclude a small number of flies whose behavior and/or imaging degraded in quality, a known limitation of fly-on-a-ball calcium imaging experiments.
Replication	We specifically focused on the peak performance of the internal compass of flies walking in unnatural settings (on a ball), highlighting the top two performers. We additionally report performance across all flies as well. Given the variability and constraints of the experiment discussed above, we do not expect the performance of the internal compass to be perfectly accurate for all flies. In addition, in response to reviewer comments, we collected data from a new set of 10 flies with longer trial durations than were presented to flies featured in our initial submission: the main results replicated across these two different groups.
Randomization	Not relevant. Data was only collected from one genotype of flies.
Blinding	Not relevant. Data was only collected from one genotype of flies.

Reporting for specific materials, systems and methods

We require information from authors about some types of materials, experimental systems and methods used in many studies. Here, indicate whether each material, system or method listed is relevant to your study. If you are not sure if a list item applies to your research, read the appropriate section before selecting a response.

Materials & experimental systems

n/a	Involved in the study	
☒	☐	Antibodies
☒	☐	Eukaryotic cell lines
☒	☐	Palaeontology and archaeology
☐	☒	Animals and other organisms
☒	☐	Clinical data
☒	☐	Dual use research of concern

Methods

n/a	Involved in the study	
☒	☐	ChIP-seq
☒	☐	Flow cytometry
☒	☐	MRI-based neuroimaging

Animals and other research organisms

Policy information about studies involving animals; ARRIVE guidelines recommended for reporting animal research, and Sex and Gender in Research

Laboratory animals	All experiments featured Drosophila melanogaster females (age 5-9d) expressing GCaMP6f in EPG neurons.
Wild animals	No wild animals were used in this study.
Reporting on sex	Female flies are larger, making them more suitable for calcium imaging experiments in tethered behaving flies.
Field-collected samples	No field collected samples were used in the study.
Ethics oversight	Studies in Drosophila do not require ethical approval. Treatment of flies was in accordance with standard lab procedures.

Note that full information on the approval of the study protocol must also be provided in the manuscript.
